# Mental Health Crisis Among Married Women in Bangladesh: Risk Factors and the Role of Misdiagnosis

**DOI:** 10.1155/da/5515696

**Published:** 2026-08-30

**Authors:** Jahar Bhowmik, Lakma Gunarathne, Raaj Kishore Biswas

**Affiliations:** ^1^ Department of Biomedical, Health and Exercise Sciences, School of Health Sciences, Swinburne University of Technology, Hawthorn VIC 3122, Melbourne, Australia, swinburne.edu.au; ^2^ Charles Perkins Centre, Faculty of Medicine and Health, The University of Sydney, Sydney, New South Wales, Australia, sydney.edu.au; ^3^ Snow Vision Accelerator, Save Sight Institute, The University of Sydney, Sydney, New South Wales, Australia, sydney.edu.au; ^4^ Clinical Research Centre, Sydney Local Health District, Sydney, New South Wales, Australia, nsw.gov.au

**Keywords:** anxiety, autonomy, depression, intimate partner violence, mental health, spatial analysis

## Abstract

**Background:**

In Bangladesh, women experience common mental health conditions like depression and anxiety at twice the rate of men, yet they are only half as likely to seek treatment. There were instances of misdiagnosis reported among married women. This study aims to assess the prevalence, key determinants and diagnostic accuracy of anxiety and depression among married women in Bangladesh.

**Methodology:**

We used the largest cross‐sectional nationally representative Bangladesh Demographic and Health Survey (BDHS) 2022 that sampled 18,605 married women, aged 15–49 years. We utilised complex survey‐adjusted binary and multinomial logistic regression models to explore the association between mental health diagnoses and sociodemographic factors.

**Results:**

One in three women reported symptoms of anxiety and depression without a clinical diagnosis. Significant regional variation was observed, with women in Dhaka, Mymensingh and Rajshahi less likely to be symptomatic but undiagnosed. Protective factors included higher education, internet access, and household wealth, while media exposure, mobile phone ownership, and attitudes accepting violence increased risk. Women accepting violence had 1.18 times (95% CI = 1.07–1.30) higher odds of being symptomatic but undiagnosed. Those involved in household decision‐making had 26% (95% CI = 1.14–1.40) higher odds compared to women who were correctly identified. In contrast, women with secondary and higher education had 17% (95% CI = 0.73–0.95) and 20% (95% CI = 0.66–0.97) lower odds, respectively.

**Conclusion:**

Targeted interventions through health literacy interventions are needed in regions with higher rates of symptomatic but undiagnosed cases. Awareness campaigns should also address the harmful effects of media exposure, limited digital access and social norms that normalise violence.

## 1. Introduction

Mental health conditions overall rank as the sixth leading contributor to the global burden of disease [1]. As of 2020, nearly 1 billion people worldwide suffer from a mental illness, and one person dies by suicide every 40 s [2–4]. Mental health is fundamental to both individual and community well‐being. Women’s mental health is vital for their own and familial well‐being. In low‐ and middle‐income countries (LMICs), such as Bangladesh, mental health remains neglected due to its lack of apparent urgency compared to more visible physical health concerns and the limited allocation of resources toward mental health services.

In Bangladesh, women are more likely than men to experience mental disorders, with anxiety and depression being the most prevalent, particularly among married women [5]. It is reported that 18.8% of women experience mental health disorders, with depression, anxiety, and stress being the most common [6].

Socioeconomic disadvantage is a fundamental driver of mental health outcomes across the life course [7, 8]. Clear socioeconomic gradients have been consistently documented for a wide range of mental health outcomes in both high‐income countries and LMIC settings [7, 9]. As a multifaceted construct, socioeconomic disadvantage can be conceptualised in several ways, encompassing interconnected dimensions such as educational attainment, financial resources, occupational status and living standards [7, 10]. The main factors contributing to mental health challenges faced by women include financial status, limited decision‐making autonomy, exposure to interpersonal violence and societal expectations related to their roles as child bearers and caregivers [11–13]. Between 2001 and 2016, data from the country’s only government mental hospital showed that around two‐thirds of the patients were male, despite national data indicating a higher prevalence of mental disorders among women—a disparity the authors attributed to low awareness and social stigma [14]. Gender equality and decision‐making power have been shown to be associated with mental health outcomes, particularly among women. Previous studies suggest that women are more likely than men to experience food insecurity and that severe food insecurity among women is linked to an increased risk of mental health diagnoses [15].

Beyond material deprivation, women’s empowerment and geographic context are important determinants of mental health. Limited household decision‐making power, restricted mobility and low economic participation have been associated with higher risks of anxiety and depression among women in South Asia, including Bangladesh [16, 17]. Greater empowerment—reflected in financial autonomy and participation in household decisions—appears to buffer psychological distress by increasing agency and access to support [18, 19]. Regional disparities persist within Bangladesh, driven by unequal access to healthcare, social mobility, and cultural practices, with women in disadvantaged or remote divisions experiencing compounded mental health risks [20].

In Bangladesh, women face several barriers to accessing mental health services, including limited mental health literacy, stigma surrounding the seeking of mental healthcare, the inadequate availability of mental health services and social isolation experienced by those living with mental illness [6, 6, 21]. As a result, mental health disorders such as anxiety and depression often remain underdiagnosed or misdiagnosed. There is a lack of systematic understanding on the relationship between self‐reported anxiety and depressive symptoms and formally diagnosed disorders assessed using validated scales [22–25].

In Bangladesh, where mental health literacy is low and primary healthcare services often lack specialised mental health screening tools [14, 26, 27], understanding the factors associated with diagnostic inaccuracy is crucial for improving mental health interventions and policies. However, these factors have not been systematically examined in previous research. Moreover, the majority of studies conducted in Bangladesh have relied on locally collected data, lacking a national representative scope. Our study aimed to assess the prevalence and examine the socioeconomic determinants of anxiety and depression and its diagnostic accuracy through verbal autopsy among married women in Bangladesh. By investigating the factors associated with symptomatic but undiagnosed or asymptomatic of mental health conditions using the most up‐to‐date national level representative Bangladesh Demographic and Health Survey (BDHS) 2022 data, this study aimed to address critical gaps in diagnostic accuracy and contribute to the development of more effective mental health strategies.

## 2. Method

### 2.1. Data Source and Study Design

The current study utilised de‐identified secondary data from the latest available BDHS, conducted from May to July 2022. The BDHS is a nation‐wide cross‐sectional survey which use two‐stage stratified cluster sampling. The primary sampling units are the enumeration areas (EAs) considered from the census data. In the first stage of the survey methodology, a stratified sample of 675 EAs (known as clusters) was selected using the probability‐proportional‐to‐size sampling technique. A pre‐determined number of households (45 households) are selected from EAs using an equal‐probability systematic‐sampling method at the second stage, resulting in a total of 30,330 residential households (19,665 from rural areas and 10,665 from urban areas). At the final stage, after administering a household questionnaire in each household, an individual survey is conducted, interviewing every eligible woman aged 15–49 years from that household [28].

#### 2.1.1. Participants and Samples

The final cleaned analytical dataset for this study included a total sample of 18,605 married women aged 15–49 years.

### 2.2. Variables

#### 2.2.1. Outcome Variables

This study considered four outcome variables: self‐reported anxiety, self‐reported depression, self‐reported overall mental health, and mental health diagnosis. Self‐reported anxiety and depression were assessed using the Generalized Anxiety Disorder‐7 (GAD‐7) scale [29] and the Patient Health Questionnaire‐9 (PHQ‐9) [30], both of which are validated self‐reported screening tools. Details on the items, scoring methods, and classification thresholds for the GAD‐7 and PHQ‐9 scales are provided in Supporting Information S2 (Section S1).

To assess the self‐reported overall mental health, a merged variable named ‘self‐reported mental health’ was created, which classified participants as having self‐reported mental health issues if they met the threshold for either mild‐to‐severe anxiety (GAD‐7 score ≥ 5) or mild‐to‐severe depression (PHQ‐9 score ≥ 5); otherwise, they were classified as having no self‐reported mental health issues [29, 30].

The mental health diagnosis variable was simulated to assess differences between self‐reported (via GAD‐7 and PHQ‐9) and clinically diagnosed mental health conditions. In this study, clinical diagnosis was based on participants’ responses to whether they had ever been told by a doctor or healthcare provider that they had anxiety or depression. Participants were then categorised into three distinct groups based on the combination of self‐reported mental health and reported clinical diagnoses. The first category, ‘correctly identified’, included participants whose self‐reported mental health status aligned with their reported clinical diagnosis (i.e., whether participants did or did not report symptoms of anxiety or depression on the GAD‐7 or PHQ‐9 aligned with whether they had or had not been clinically diagnosed with a mental health condition by a healthcare provider). The second category, ‘Symptomatic but Undiagnosed’, included participants who self‐reported symptoms of anxiety or depression but had not been clinically diagnosed by a healthcare provider. The third category, ‘Asymptomatic’, included participants who were clinically diagnosed with anxiety or depression but did not report symptoms of anxiety or depression based on the GAD‐7 or PHQ‐9 self‐reported screening tools.

#### 2.2.2. Conceptual Framework for Explanatory Variables

**Figure 1:**
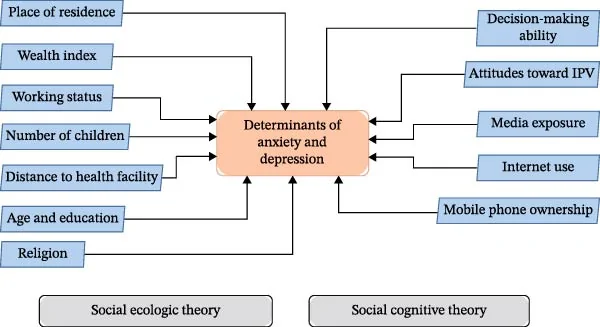
Conceptual framework illustrating factors associated with anxiety and depression based on Social Ecological Theory and Social Cognitive Theory.

Multiple theoretical perspectives explain the factors associated with mental health outcomes. Among these, the social ecological theory and the cognitive theory provide a useful foundation for understanding the determinants of anxiety and depression. This study is therefore guided by these two complementary frameworks, as illustrated in Figure 1. The Social Ecological Theory suggests that mental health is shaped by factors operating at multiple levels, including individual, household, and broader contextual environments [31, 32]. Accordingly, this study considers sociodemographic characteristics, household conditions, and contextual factors as key determinants of anxiety and depression. Individual sociodemographic characteristics, such as age, education, religion and age at first cohabitation, may influence mental health through differences in life experience, knowledge and vulnerability to stressors. Household‐level factors, including wealth status, employment, number of children, and access to healthcare, reflect economic conditions and caregiving responsibilities that may shape exposure to stress and access to resources. Contextual factors such as place of residence and geographic region capture environmental influences that may affect mental health through variations in social norms, service availability and living conditions.

The social cognitive theory further explains how these factors influence mental health through cognitive and behavioural pathways [33]. According to this theory, individuals’ beliefs, perceived control, and exposure to information shape their ability to cope with stress and maintain psychological well‐being. In this study, women’s empowerment, measured through decision‐making ability and attitudes toward intimate partner violence (IPV), reflects perceived autonomy and control over life circumstances. Similarly, media exposure, internet use, and mobile phone ownership may influence mental health by shaping knowledge, social connectivity and the cognitive response to stress. Together, these theories provide a multi‐level and mechanism‐based framework for understanding mental health outcomes in the study population (Figure 1).

#### 2.2.3. Explanatory Variables

Based on the conceptual framework, past literature, and the availability of variables in the BDHS dataset [34–37], 18 explanatory variables were selected. All variables were extracted from DHS datasets and used according to the definitions provided in the BDHS report [28]. Geographic variables included administrative division (Barisal, Chittagong, Dhaka, Khulna, Mymensingh, Rajshahi, Rangpur and Sylhet) and area of residence (urban and rural). Sociodemographic and household characteristics variables included religion (Islam, other), education levels of both respondent and her partner (no education, primary, secondary, higher), owns a mobile phone (no, yes), media exposure (not exposed, exposed TV, radio, and/or newspapers), ever use of internet (no, yes), media exposure (not exposed, exposed TV, radio, and/or newspapers), ever use of internet (no, yes), number of children ever born (0 or 1 child, 2 children, 3 children, 4 or more children), both respondent and her partner’s working status (not working, working), distance to a health facility (big problem, not a big problem), both respondent and her partner’s age (in years), and respondent’s age at first cohabitation (in years). Household socioeconomic status was measured using the DHS wealth index, a standard composite variable provided in the BDHS dataset. The wealth index was constructed by DHS using principal component analysis based on household assets and living conditions. The resulting score is categorised into five nationally representative wealth quintiles: poorest, poorer, middle, richer, and richest. These categories were used directly, as provided in the dataset. Women’s empowerment was assessed using attitudes toward IPV, recoded as a binary variable where a woman was categorised as ‘Yes’ if she agreed it was acceptable for a husband to hit his wife in any of five hypothetical scenarios: going out without telling partner, neglecting children, arguing, refusing sex, or burning food; and ‘No’ if none were agreed with and respondent’s decision‐making ability, recoded as a binary variable as ‘Does not make decisions’ and ‘Makes decisions’, where ‘Makes decisions’ indicates involvement in at least one of the following decisions her own health care, major household purchases, and/or visits to family or relatives. The survey weight was also extracted for modelling purposes.

### 2.3. Statistical Analysis

Missing values were removed list‐wise assuming that data were missing completely at random [38, 39]. Unweighted bivariate analyses were conducted to explore the distribution of sociodemographic variables across the outcome variables. Associations between outcome variables and categorical sociodemographic variables were assessed using the Chi‐square test, while independent sample *t*‐test and ANOVA were used for continuous variables, depending on the number of categories. Prior to conducting parametric tests, the homogeneity of variances was assessed using Levene’s test. For independent‐samples *t*‐tests, the equal variances assumed test was used when Levene’s test was not significant; otherwise, Welch’s *t*‐test was applied. For one‐way ANOVA, Tukey’s HSD test was used for post hoc pairwise comparisons when the assumption of equal variances was satisfied, whereas Games–Howell post hoc comparisons were applied when the homogeneity of variances was violated. These analyses were exploratory in nature. Given the large sample size, parametric tests were considered appropriate. To examine the direction and effect of adjusted association between sociodemographic variables with the self‐reported anxiety, depression, and mental health, three separate complex survey adjusted logistic regression models were fitted with additional adjustment for survey weights, strata, and cluster. As the mental health diagnosis variable had three categories, a fully adjusted multinominal logistic regression model was fitted to assess the associations between mental health diagnosis and sociodemographic variables, accounting for the complex survey design, including strata, clusters, and sampling weights. Explanatory variables were selected based on the conceptual framework, previous literature, objective of the study, and availability in the BDHS dataset. All selected variables were entered simultaneously into the adjusted models to estimate associations while controlling for potential confounding. Multicollinearity among explanatory variables was assessed using generalized variance inflation factors (GVIFs), with adjusted GVIF values reported for multi‐level categorical variables [40]. No evidence of problematic multicollinearity was observed. The complete GVIF results are presented in Table S2 of Supporting Information S2. In this study, we applied a significance level with a *p*‐value threshold of <0.05. All analyses were conducted in SPSS (v.29) and R (v.4.3.2).

## 3. Results

The analytic sample included 18,605 married women (mean age 31.7 ± 8.8 years; Table 1). Clinically diagnosed anxiety and depression were 6.8% and 4.1%, respectively, while self‐reported GAD‐7 and PHQ‐9 assessments showed higher rates: 25.6% for anxiety and 28.2% for depression. Overall, 65.9% were correctly identified, 32% were symptomatic but undiagnosed, and 2.1% were asymptomatic. Most participants were Muslim (89.6%), lived rurally (65.1%), experienced interpersonal violence (86.7%), and had decision‐making autonomy (85.1%).

**Table 1:** Sociodemographic characteristics of the married women in the survey (*N* = 18,605).

Variables	*N*/mean	Percentages/SD
Division		
Barisal	2004	10.8
Chittagong	2757	14.8
Dhaka	2788	15.0
Khulna	2444	13.1
Mymensingh	2023	10.9
Rajshahi	2405	12.9
Rangpur	2227	12.0
Sylhet	1957	10.5
Religion		
Islam	16,675	89.6
Other	1930	10.4
Area of residence		
Urban	6485	34.9
Rural	12,120	65.1
Level of education		
No education	2373	12.8
Primary	4799	25.8
Secondary	8630	46.4
Higher	2803	15.1
Owns a mobile telephone		
No	5718	30.7
Yes	12,887	69.3
Media exposure		
Not exposed	7677	41.3
Exposed	10,928	58.7
Ever use internet		
No	13,095	70.4
Yes	5510	29.6
Wealth index		
Poorest	3300	17.7
Poorer	3638	19.6
Middle	3693	19.8
Richer	3872	20.8
Richest	4102	22.0
Number of children		
No or 1 child	6062	32.6
2 children	6093	32.7
3 children	3761	20.2
4 or more children	2689	14.5
Current working status		
Not working	12,982	69.8
Working	5623	30.2
Attitudes toward violence		
No	16,129	86.7
Yes	2476	13.3
Decision making power		
Does not make decisions	2772	14.9
Makes decisions	15,833	85.1
Husband’s level of education		
No education	3918	21.1
Primary	5218	28.0
Secondary	5980	32.1
Higher	3489	18.8
Husband’s working status		
Not working	560	3.0
Working	18,045	97.0
Distance to health facility		
Big problem	8453	45.4
Not a big problem	10,152	54.6
Anxiety (clinically diagnosed)		
No	17,345	93.2
Yes	1260	6.8
Depression (clinically diagnosed)		
No	17,839	95.9
Yes	766	4.1
Self‐reported Anxiety (based on GAD‐7)		
No anxiety	13,837	74.4
Anxiety	4768	25.6
Self‐reported depression (based on PHQ‐9)		
No depression	13,350	71.8
Depression	5255	28.2
Mental health diagnosis		
Correctly identified	12,262	65.9
Symptomatic but undiagnosed	5950	32.0
Asymptomatic	393	2.1
Respondent’s age^a^	31.74	8.83
Age at first cohabitation^a^	16.75	3.63
Husband’s age^a^	39.7	10.63

^a^Metric variables.

**Figure 2:**
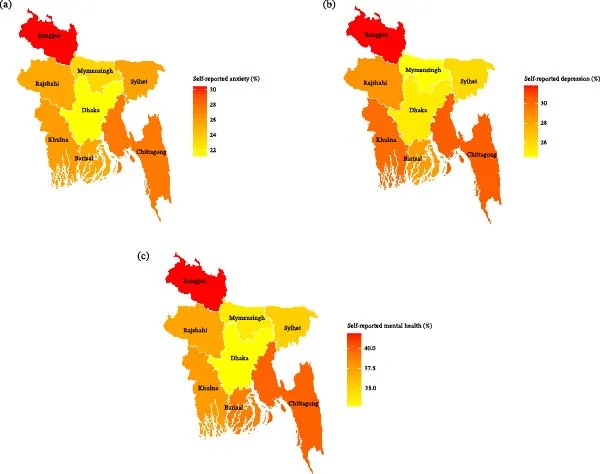
Prevalence of self‐reported anxiety, depression, and overall mental health issues among married women across divisions in Bangladesh. (a) Self‐reported anxiety (GAD‐7), (b) self‐reported depression (PHQ‐9) and (c) overall self‐reported mental health prevalence based on combined anxiety and depression symptoms.

Figure 2 illustrates regional variation in the prevalence of self‐reported anxiety and depression among married women across Bangladesh. Anxiety prevalence (GAD‐7) was highest in Rangpur (30.4%), followed by Chattogram (27.6%) and Khulna (26.1%). Lower prevalence was observed in Dhaka (21.2%) and Mymensingh (23.1%). A similar pattern was observed for depression (PHQ‐9). The prevalence of depression was highest in Rangpur (31.8%), followed by Chattogram (30.4%), and Khulna (30.0%). Lower prevalence was observed in Mymensingh (24.6%) and Dhaka (25.4%). Overall, the combined prevalence of self‐reported mental health issues (anxiety or depression) was highest in Rangpur (41.9%) and Chattogram (40.0%), followed by Barisal (38.9%) and Khulna (37.7%), while lower prevalence was observed in Dhaka (32.9%) and Mymensingh (33.9%).

**Figure 3:**
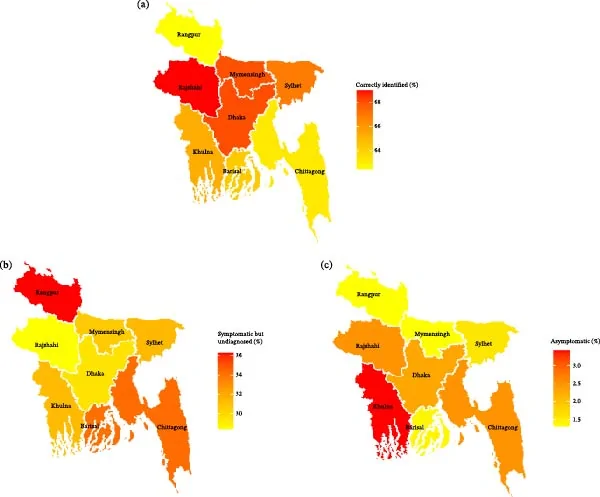
Spatial variation in mental health diagnosis categories among married women across divisions in Bangladesh. (a) Correctly identified, (b) symptomatic but undiagnosed and (c) asymptomatic mental health status.

Figure 3 illustrates regional variation in mental health diagnosis categories among married women across divisions in Bangladesh. The prevalence of correctly identified mental health status was highest in Rajshahi (69.0%) and Mymensingh (68.0%), followed by Dhaka (68.1%) and Sylhet (66.9%), while lower levels were observed in Chattogram (63.2%) and Rangpur (62.4%). For symptomatic but undiagnosed cases, Rangpur reported the highest prevalence (36.3%), followed by Chattogram (34.3%) and Barisal (34.2%). Lower prevalence was observed in Rajshahi (28.4%) and Dhaka (29.6%). For asymptomatic cases (clinically diagnosed but not reporting symptoms), Khulna recorded the highest prevalence (3.4%), followed by Rajshahi (2.5%) and Chattogram (2.5%), while lower levels were observed in Rangpur (1.3%) and Mymensingh (1.3%).

The prevalence of anxiety and depression diagnoses across divisions is presented separately in Figures S1 and S2 of Supporting Information S2, respectively.

Table 2 shows unweighted bivariate associations between sociodemographic factors and women’s self‐reported mental health outcomes (anxiety, depression and overall mental health) and diagnostic accuracy. Women of non‐Muslim faith reported lower anxiety (20.5%) than Muslim women (26.5%), with similar trends for depression and overall mental health. Diagnosis was more accurate among non‐Muslims (70.4% vs. 65.4%), while symptomatic but undiagnosed was higher among Muslims (32.5% vs. 27.8%).

**Table 2:** Bi‐variate (unweighted) associations of women’s self‐reported mental health outcomes (anxiety, depression and mental health) and mental health diagnosis with the selected sociodemographic variables; *N* = 18,605.

Variables	Self‐reported anxiety		Self‐reported depression		Self‐reported mental health		Mental health diagnosis		
	No anxiety (*N* = 13,837, 74.4%)	Anxiety (*N* = 4768, 25.6%)	No depression (*N* = 13350, 71.8%)	Depression (*N* = 5255, 28.2%)	Healthy (*N* = 11,683, 62.8%)	Not healthy (*N* = 6922, 37.2%)	Correctly Identified (*N* = 12,262, 65.9%)	Symptomatic but undiagnosed (*N* = 5950, 32.0%)	Asymptomatic (*N* = 393, 2.1%)
	*N* (%)	*N* (%)	*N* (%)	*N* (%)	*N* (%)	*N* (%)	*N* (%)	*N* (%)	*N* (%)
Division									
Barisal	1490 (74.4)	514 (25.6)	1434 (71.6)	570 (28.4)	1224 (61.1)	780 (38.9)	1289 (64.3)	686 (34.2)	29 (1.5)
Chittagong	1997 (72.4)	760 (27.6)	1919 (69.6)	838 (30.4)	1654 (60)	1103 (40)	1742 (63.2)	947 (34.3)	68 (2.5)
Dhaka	2196 (78.8)	592 (21.2)	2080 (74.6)	708 (25.4)	1872 (67.1)	916 (32.9)	1900 (68.1)	825 (29.6)	63 (2.3)
Khulna	1806 (73.9)	638 (26.1)	1712 (70)	732 (30)	1523 (62.3)	921 (37.7)	1596 (65.3)	764 (31.3)	84 (3.4)
Mymensingh	1555 (76.9)	468 (23.1)	1526 (75.4)	497 (24.6)	1337 (66.1)	686 (33.9)	1375 (68.0)	621 (30.7)	27 (1.3)
Rajshahi	1790 (74.4)	615 (25.6)	1714 (71.3)	691 (28.7)	1510 (62.8)	895 (37.2)	1660 (69.0)	684 (28.4)	61 (2.5)
Rangpur	1551 (69.6)	676 (30.4)	1519 (68.2)	708 (31.8)	1295 (58.1)	932 (41.9)	1390 (62.4)	808 (36.3)	29 (1.3)
Sylhet	1452 (74.2)	505 (25.8)	1446 (73.9)	511 (26.1)	1268 (64.8)	689 (35.2)	1310 (66.9)	615 (31.4)	32 (1.6)
*p*‐Value (Cramer’s *V*)	*<0.001* (*0.060*)		*<0.001* (*0.053*)		*<0.001* (*0.060*)		*<0.001* (*0.051*)		
Religion									
Islam	12,302 (73.8)	4373 (26.2)	11,877 (71.2)	4798 (28.8)	10,359 (62.1)	6316 (37.9)	10,904 (65.4)	5414 (32.5)	357 (2.1)
Other	1535 (79.5)	395 (20.5)	1473 (76.3)	457 (23.7)	1324 (68.6)	606 (31.4)	1358 (70.4)	536 (27.8)	36 (1.9)
*p*‐Value (Cramer’s *V*)	*<0.001* (*0.040*)		*<0.001* (*0.035*)		*<0.001* (*0.041*)		*<0.001* (*0.032*)		
Area of residence									
Urban	4855 (74.9)	1630 (25.1)	4648 (71.7)	1837 (28.3)	4091 (63.1)	2394 (36.9)	4265 (65.8)	2075 (32.0)	145 (2.2)
Rural	8982 (74.1)	3138 (25.9)	8702 (71.8)	3418 (28.2)	7592 (62.6)	4528 (37.4)	7997 (66.0)	3875 (32.0)	248 (2.0)
*p*‐Value (Cramer’s *V*)	*0.260* (*0.008*)		*0.856* (*0.001*)		*0.551* (*0.004*)		*0.687* (*0.006*)		
Level of education									
No education	1554 (65.5)	819 (34.5)	1568 (66.1)	805 (33.9)	1290 (54.4)	1083 (45.6)	1408 (59.3)	921 (38.8)	44 (1.9)
Primary	3398 (70.8)	1401 (29.2)	3332 (69.4)	1467 (30.6)	2837 (59.1)	1962 (40.9)	3016 (62.8)	1688 (35.2)	95 (2.0)
Secondary	6608 (76.6)	2022 (23.4)	6319 (73.2)	2311 (26.8)	5618 (65.1)	3012 (34.9)	5867 (68)	2584 (29.9)	179 (2.1)
Higher	2277 (81.2)	526 (18.8)	2131 (76.0)	672 (24.0)	1938 (69.1)	865 (30.9)	1971 (70.3)	757 (27.0)	75 (2.7)
*p*‐Value (Cramer’s *V*)	*<0.001* (*0.109*)		*<0.001* (*0.068*)		*<0.001* (*0.095*)		*<0.001* (*0.058*)		
Owns a mobile telephone									
No	4230 (74.0)	1488 (26.0)	4024 (70.4)	1694 (29.6)	3548 (62.0)	2170 (38.0)	3707 (64.8)	1899 (33.2)	112 (2.0)
Yes	9607 (74.5)	3280 (25.5)	9326 (72.4)	3561 (27.6)	8135 (63.1)	4752 (36.9)	8555 (66.4)	4051 (31.4)	281 (2.2)
*p*‐Value (Cramer’s *V*)	*0.410* (*0.006* **)**		*0.005* (*0.020*)		*0.161* (*0.010*)		*0.043* (*0.018*)		
Media exposure									
Not exposed	5623 (73.2)	2054 (26.8)	5576 (72.6)	2101 (27.4)	4826 (62.9)	2851 (37.1)	5076 (66.1)	2447 (31.9)	154 (2.0)
Exposed	8214 (75.2)	2714 (24.8)	7774 (71.1)	3154 (28.9)	6857 (62.7)	4071 (37.3)	7186 (65.8)	3503 (32.1)	239 (2.2)
*p*‐Value (Cramer’s *V*)	*0.003* (*0.022*)		*0.026* (*0.016*)		*0.872* (*0.001*)		*0.659* (*0.007*)		
Ever use of internet									
No	9458 (72.2)	3637 (27.8)	9212 (70.3)	3883 (29.7)	7932 (60.6)	5163 (39.4)	8403 (64.2)	4430 (33.8)	262 (2.0)
Yes	4379 (79.5)	1131 (20.5)	4138 (75.1)	1372 (24.9)	3751 (68.1)	1759 (31.9)	3859 (70.0)	1520 (27.6)	131 (2.4)
*p*‐Value (Cramer’s *V*)	*<0.001* (*0.076*)		*<0.001* (*0.048*)		*<0.001* (*0.071*)		*<0.001* (*0.061*)		
Wealth index									
Poorest	2331 (70.6)	969 (29.4)	2321 (70.3)	979 (29.7)	1972 (59.8)	1328 (40.2)	2082 (63.1)	1163 (35.2)	55 (1.7)
Poorer	2615 (71.9)	1023 (28.1)	2556 (70.3)	1082 (29.7)	2203 (60.6)	1435 (39.4)	2357 (64.8)	1210 (33.3)	71 (2.0)
Middle	2749 (74.4)	944 (25.6)	2635 (71.4)	1058 (28.6)	2307 (62.5)	1386 (37.5)	2415 (65.4)	1191 (32.3)	87 (2.4)
Richer	2902 (74.9)	970 (25.1)	2780 (71.8)	1092 (28.2)	2450 (63.3)	1422 (36.7)	2598 (67.1)	1204 (31.1)	70 (1.8)
Richest	3240 (79.0)	862 (21.0)	3058 (74.5)	1044 (25.5)	2751 (67.1)	1351 (32.9)	2810 (68.5)	1182 (28.8)	110 (2.7)
*p*‐Value (Cramer’s *V*)	*<0.001* (*0.067*)		*<0.001* (*0.035*)		*<0.001* (*0.054*)		*<0.001* (*0.036*)		
Number of children									
No or 1 child	4829 (79.7)	1233 (20.3)	4559 (75.2)	1503 (24.8)	4140 (68.3)	1922 (31.7)	4241 (70.0)	1702 (28.1)	119 (2.0)
2 children	4570 (75.0)	1523 (25.0)	4405 (72.3)	1688 (27.7)	3862 (63.4)	2231 (36.6)	4062 (66.7)	1913 (31.4)	118 (1.9)
3 children	2666 (70.9)	1095 (29.1)	2608 (69.3)	1153 (30.7)	2224 (59.1)	1537 (40.9)	2372 (63.1)	1308 (34.8)	81 (2.2)
≥4 children	1772 (65.9)	917 (34.1)	1778 (66.1)	911 (33.9)	1457 (54.2)	1232 (45.8)	1587 (59.0)	1027 (38.2)	75 (2.8)
*p*‐Value (Cramer’s *V*)	*<0.001* (*0.108*)		*<0.001* (*0.069*)		*<0.001* (*0.100*)		*<0.001* (*0.056*)		
Working Status									
Not working	9737 (75.0)	3245 (25.0)	9418 (72.5)	3564 (27.5)	8244 (63.5)	4738 (36.5)	8618 (66.4)	4099 (31.6)	265 (2)
Working	4100 (72.9)	1523 (27.1)	3932 (69.9)	1691 (30.1)	3439 (61.2)	2184 (38.8)	3644 (64.8)	1851 (32.9)	128 (2.3)
*p*‐Value (Cramer’s *V*)	*0.003* (*0.022*)		*<0.001* (*0.027*)		*0.002* (*0.022*)		*0.094* (*0.016*)		
Attitudes toward violence									
No	12,145 (75.3)	3984 (24.7)	11,681 (72.4)	4448 (27.6)	10,275 (63.7)	5854 (36.3)	10,722 (66.5)	5060 (31.4)	347 (2.2)
Yes	1692 (68.3)	784 (31.7)	1669 (67.4)	807 (32.6)	1408 (56.9)	1068 (43.1)	1540 (62.2)	890 (35.9)	46 (1.9)
*p*‐Value (Cramer’s *V*)	*<0.001* (*0.054*)		*<0.001* (*0.038*)		*<0.001* (*0.048*)		*<0.001* (*0.033*)		
Decision‐making ability									
Does not make decisions	2159 (77.9)	613 (22.1)	2090 (75.4)	682 (24.6)	1870 (67.5)	902 (32.5)	1960 (70.7)	769 (27.7)	43 (1.6)
Makes decisions	11,678 (73.8)	4155 (26.2)	11,260 (71.1)	4573 (28.9)	9813 (62.0)	6020 (38.0)	10,302 (65.1)	5181 (32.7)	350 (2.2)
*p*‐Value (Cramer’s *V*)	*<0.001* (*0.034*)		*<0.001* (*0.034*)		*<0.001* (*0.040*)		*<0.001* (*0.043*)		
Husband’s education level									
No education	2643 (67.5)	1275 (32.5)	2670 (68.1)	1248 (31.9)	2211 (56.4)	1707 (43.6)	2395 (61.1)	1438 (36.7)	85 (2.2)
Primary	3829 (73.4)	1389 (26.6)	3707 (71)	1511 (29)	3226 (61.8)	1992 (38.2)	3368 (64.5)	1744 (33.4)	106 (2)
Secondary	4546 (76)	1434 (24)	4328 (72.4)	1652 (27.6)	3857 (64.5)	2123 (35.5)	4056 (67.8)	1814 (30.3)	110 (1.8)
Higher	2819 (80.8)	670 (19.2)	2645 (75.8)	844 (24.2)	2389 (68.5)	1100 (31.5)	2443 (70.0)	954 (27.3)	92 (2.6)
*p*‐Value (Cramer’s *V*)	*<0.001* (*0.100*)		*<0.001* (*0.055*)		*<0.001* (*0.082*)		*<0.001* (*0.050*)		
Husband’s working status									
Not working	359 (64.1)	201 (35.9)	370 (66.1)	190 (33.9)	312 (55.7)	248 (44.3)	346 (61.8)	207 (37.0)	7 (1.3)
Working	13,478 (74.7)	4567 (25.3)	12,980 (71.9)	5065 (28.1)	11,371 (63.0)	6674 (37.0)	11,916 (66.0)	5743 (31.8)	386 (2.1)
*p*‐Value (Cramer’s *V*)	*<0.001* (*0.041*)		*0.002* (*0.022*)		*<0.001* (*0.026*)		*0.018* (*0.021*)		
Distance to health facility									
Big problem	5942 (70.3)	2511 (29.7)	5784 (68.4)	2669 (31.6)	4948 (58.5)	3505 (41.5)	5315 (62.9)	2971 (35.1)	167 (2.0)
Not a big problem	7895 (77.8)	2257 (22.2)	7566 (74.5)	2586 (25.5)	6735 (66.3)	3417 (33.7)	6947 (68.4)	2979 (29.3)	226 (2.2)
*p*‐Value (Cramer’s *V*)	*<0.001* (*0.085*)		*<0.001* (*0.067*)		*<0.001* (*0.080*)		*<0.001* (*0.062*)		
	**Mean (SD)**	**Mean (SD)**	**Mean (SD)**	**Mean (SD)**	**Mean (SD)**	**Mean (SD)**	**Mean (SD)**	**Mean (SD)**	**Mean (SD)**
Age^a^	31.13 (8.83)	33.52 (8.58)	31.30 (8.81)	32.86 (8.77)	31.01 (8.83)	32.98 (8.69)	31.24 (8.85)	32.68 (8.69)	33.09 (8.91)
*p*‐Value (Cohen’s *d/η* ^2^)	*<0.001* (*0.274*)		*<0.001* (*0.178*)		*<0.001* (*0.224*)		*<0.001* (*0.006*)		
Age at first cohabitation^a^	16.84 (3.58)	16.51 (3.77)	16.81 (3.58)	16.62 (3.76)	16.85 (3.56)	16.59 (3.74)	16.8 (3.57)	16.64 (3.75)	17.1 (3.64)
*p*‐Value (Cohen’s *d/η* ^2^)	*<0.001* (*0.090*)		*<0.001* (*0.052*)		*<0.001* (*0.073*)		*0.004* (*0.001*)		
Husband’s age ^a^	39.01 (10.53)	41.73 (10.67)	39.22 (10.54)	40.94 (10.77)	38.89 (10.52)	41.07 (10.67)	39.17 (10.57)	40.72 (10.68)	40.94 (10.47)
*p*‐Value (Cohen’s *d/η* ^2^)	*<0.001* (*0.258*)		*<0.001* (*0.162*)		*<0.001* (*0.206*)		*<0.001* (*0.005*)		

*Note:* Italic values represent the *p*‐values and corresponding effect size measures (Cramer’s *V* or Cohen’s *d/η* ^2^) and are used to distinguish these statistical test results from the descriptive statistics presented in regular font.

^a^Metric variables.

The education level showed strong associations. Women with no formal education had higher rates of anxiety (34.5%) and depression (33.9%) compared to those with higher education (18.8% and 24%, respectively). Diagnosis accuracy improved with education (70.3% vs. 59.3%), while symptomatic but undiagnosed was more frequent among uneducated women (38.8%) than those with higher education (27%). Similar patterns were observed based on the husbands’ education levels.

Mobile phone ownership also showed modest differences: women without phones had higher depression rates (29.6% vs. 27.6%) and slightly more frequent symptomatic but undiagnosed (33.2% vs. 31.4%).

Among women not exposed to the media, 26.8% reported anxiety and 27.4% reported depression. In contrast, media‐exposed women had slightly lower anxiety (24.8%) but higher depression (28.9%). Internet use was associated with better outcomes; users reported lower anxiety (20.5%) and depression (24.9%) than non‐users (27.8% and 29.7%, respectively). Diagnosis accuracy was also higher among internet users (70.0%) versus non‐users (64.2%), with lower symptomatic but undiagnosed (27.6% vs. 33.8%).

A wealth gradient was evident: women in the poorest quintile had the highest anxiety (29.4%) and depression (29.7%) rates, while the richest had the lowest (21.0% and 25.5%, respectively). Poor mental health was more common among the poorest (40.2%) than among the richest (32.9%). Diagnosis accuracy improved with wealth (63.1% vs. 68.5%), while symptomatic but undiagnosed decreased (35.2% vs. 28.8%).

Women with four or more children reported the highest anxiety (34.1%) and depression (33.9%), compared to much lower rates in those with no or one child (20.3% and 24.8%). Diagnosis accuracy declined with more children (70.0% in the no/one‐child group vs. 59.0% in the four‐or‐more group), while symptomatic but undiagnosed rose (28.1% to 38.2%).

Attitudes toward IPV were linked to worse outcomes. Women accepting IPV reported higher anxiety (31.7%) and depression (32.6%) than those who did not (24.7% and 27.6%). Diagnosis accuracy was lower among IPV‐accepting women (62.2% vs. 66.5%) and symptomatic but undiagnosed higher (35.9% vs. 31.4%). Women involved in household decision‐making also had higher anxiety (26.2%) and depression (28.9%) than those not involved (22.1% and 24.6%). Diagnosis was more accurate among non‐decision‐makers (70.7% vs. 65.1%), while symptomatic but undiagnosed was more common in decision‐makers (32.7% vs. 27.7%).

Mean respondent age, age at first cohabitation and husband’s age differed across the mental health diagnosis groups (*p*  < 0.05). Appropriate post hoc analyses were conducted following significant ANOVA results, and the detailed pairwise comparisons are presented in Supporting Information S2 (Section S2).

Detailed bivariate associations between women’s anxiety and depression diagnosis categories and the selected sociodemographic variables are presented in Table S1 of Supporting Information S2.

Compared to Barisal, women in Dhaka and Mymensingh had significantly lower odds of reporting poor mental health by 24% (95% CI: 0.63–0.91, Table 3) and 25% (95% CI: 0.61–0.93), respectively. In contrast, women in Rangpur had 22% higher odds (95% CI: 1.02–1.47) of reporting poor mental health.

**Table 3:** Results of the complex survey adjusted logistic regression models for self‐reported anxiety, self‐reported depression and self‐reported mental health after controlling for sociodemographic covariates and adjusting for survey weights (*N* = 18,605).

Factors		Self‐reported anxiety			Self‐reported depression			Self‐reported mental health		
		AOR	*p*‐Value	95% CI	AOR	*p*‐Value	95% CI	AOR	*p*‐Value	95% CI
Division (ref: Barisal)	Chittagong	1.13	0.369	0.87–1.47	1.06	0.654	0.83–1.35	1.03	0.818	0.82–1.28
	Dhaka	0.82	0.081	0.66–1.03	0.82	**0.037**	0.67–0.99	0.76	**0.002**	0.63–0.91
	Khulna	1.08	0.494	0.86–1.37	1.06	0.529	0.88–1.28	0.96	0.698	0.80–1.16
	Mymensingh	0.85	0.195	0.66–1.09	0.76	**0.020**	0.61–0.96	0.75	**0.008**	0.61–0.93
	Rajshahi	0.97	0.831	0.75–1.27	0.90	0.283	0.73–1.10	0.85	0.120	0.70–1.04
	Rangpur	**1.43**	**0.002**	1.14–1.81	**1.25**	**0.027**	1.03–1.53	**1.22**	**0.034**	1.02–1.47
	Sylhet	0.98	0.850	0.75–1.27	0.87	0.272	0.67–1.12	0.82	0.084	0.66–1.03
Area of residence (ref: Urban)	Rural	0.90	0.133	0.78–1.03	0.90	0.495	0.82–1.10	0.90	0.360	0.82–1.08
Religion (ref: Islam)	Other	**0.65**	**<0.001**	0.55–0.78	**0.70**	**<0.001**	0.58–0.85	**0.70**	**<0.001**	0.59–0.83
Level of education (ref: No education)	Primary	0.92	0.218	0.81–1.05	0.91	0.188	0.80–1.05	0.92	0.167	0.81–1.04
	Secondary	0.88	0.070	0.76–1.01	0.86	0.054	0.74–1.00	**0.85**	**0.017**	0.75–0.97
	Higher	**0.81**	**0.031**	0.66–0.98	0.84	0.075	0.68–1.02	**0.80**	**0.013**	0.67–0.96
Owns a mobile phone (ref: No)	Yes	**1.20**	**<0.001**	1.10–1.32	1.00	0.916	0.91–1.09	**1.12**	**0.009**	1.03–1.22
Media exposure (ref: Not exposed)	Exposed	**1.11**	**0.041**	1.02–1.22	**1.24**	**<0.001**	1.13–1.36	**1.19**	**<0.001**	1.08–1.30
Ever use of internet (ref: No)	Yes	**0.87**	**0.025**	0.77–0.98	0.94	0.236	0.84–1.04	**0.87**	**0.005**	0.78–0.96
Wealth index (ref: Poorest)	Poorer	0.96	0.509	0.84–1.09	0.99	0.925	0.87–1.14	0.98	0.718	0.87–1.11
	Middle	0.87	0.055	0.76–1.00	0.99	0.904	0.85–1.15	0.96	0.551	0.84–1.10
	Richer	**0.85**	**0.039**	0.73–0.99	0.91	0.254	0.78–1.07	0.90	0.137	0.77–1.04
	Richest	**0.77**	**0.003**	0.64–0.91	0.86	0.131	0.71–1.05	0.85	0.068	0.72–1.01
Number of children (ref: No or one child)	Two children	1.02	0.777	0.91–1.14	1.00	0.970	0.90–1.12	1.02	0.692	0.92–1.13
	Three children	1.08	0.254	0.95–1.22	1.06	0.366	0.93–1.21	1.10	0.107	0.98–1.24
	4 or more children	1.17	0.052	1.00–1.37	1.14	0.083	0.98–1.33	**1.21**	**0.011**	1.04–1.39
Working status (ref: Not working)	Working	0.95	0.269	0.86–1.04	1.00	0.965	0.92–1.10	0.96	0.308	0.87–1.04
Attitudes toward Violence (ref: No)	Yes	**1.30**	**<0.001**	1.15–1.46	**1.19**	**0.002**	1.07–1.34	**1.26**	**<0.001**	1.13–1.41
Decision‐making ability (ref: Does not make decision)	Makes decision	**1.18**	**0.018**	1.03–1.35	**1.20**	**0.005**	1.06–1.36	**1.22**	**0.001**	1.09–1.38
Husband’s education level (ref: No education)	Primary	0.94	0.274	0.85–1.05	1.03	0.553	0.93–1.15	0.98	0.719	0.89–1.09
	Secondary	0.89	0.079	0.78–1.01	1.01	0.821	0.90–1.15	0.93	0.198	0.83–1.04
	Higher	**0.75**	**0.002**	0.63–0.90	0.89	0.177	0.75–1.06	**0.83**	**0.022**	0.71–0.97
Husband’s working status (ref: Not working)	Working	**0.67**	**<0.001**	0.53–0.83	**0.78**	**0.026**	0.63–0.97	**0.79**	**0.025**	0.64–0.97
Distance to health facility (ref: Big problem)	Not a big problem	**0.67**	**<0.001**	0.61–0.74	**0.72**	**<0.001**	0.66–0.79	**0.69**	**<0.001**	0.63–0.76
Age^a^	—	**1.02**	**<0.001**	1.01–1.03	**1.01**	**0.025**	1.00–1.02	**1.01**	**0.002**	1.01–1.02
Age at first cohabitation^a^	—	1.00	0.862	0.99–1.01	1.01	0.451	0.99–1.02	1.00	0.672	0.99–1.01
Husband’s age^a^	—	1.01	0.179	1.00–1.01	1.00	0.369	1.00–1.01	1.00	0.307	1.00–1.01

*Note:* Bold values indicate statistically significant associations (*p* < 0.05) in the adjusted logistic regression models.

^a^Metric variables.

Non‐Muslim women were less likely to report poor mental health (30% lower odds, 95% CI: 0.59–0.83) than Muslim women. Higher educational attainment among women was associated with lower odds of poor mental health by 15% (secondary; 95% CI: 0.75–0.97) and 20% (higher; 95% CI: 0.67–0.96) compared to those with no education. However, owning a mobile phone (12% higher odds; 95% CI: 1.03–1.22) or media exposure (19% higher odds; 95% CI: 1.08–1.30) was associated with worse mental health outcomes. Conversely, internet use was protective, with 13% lower odds of poor mental health (95% CI: 0.78–0.96).

Women from richer (15%, 95% CI: 0.73–0.99) and richest (23%, 95% CI: 0.64–0.91) households had better mental health outcomes. Poor mental health was more likely among women with four or more children (21% higher odds; 95% CI: 1.04–1.39), those accepting domestic violence (26% higher odds; 95% CI: 1.13–1.41) and those involved in household decision‐making (22% higher odds; 95% CI: 1.09–1.38).

Spousal characteristics were protective: women married to highly educated husbands (17% lower odds; 95% CI: 0.71–0.97) or employed husbands (21% lower odds; 95% CI: 0.64–0.97) were less likely to report poor mental health. Lastly, not perceiving distance to health facilities as a problem was associated with a 31% reduction in the odds of poor mental health (95% CI: 0.63–0.76).

### 3.1. Symptomatic but Undiagnosed vs. Correctly Identified

Table 4 displays the results of the survey‐weight‐adjusted multinominal logistic regression model for mental health diagnosis. Compared to women from Barisal, those from Dhaka, Mymensingh and Rajshahi had 19% (95% CI: 0.68–0.95), 19% (95% CI: 0.67–0.98), and 28% (95% CI: 0.60–0.85) lower odds, respectively, of being symptomatic but undiagnosed rather than correctly identified regarding their mental health condition.

**Table 4:** Results of the multinomial regression model for mental health diagnosis, after controlling for sociodemographic covariates and adjusting for survey weights (*N* = 18,605).

Mental health diagnosis (ref: correctly identified)		Symptomatic but undiagnosed		Asymptomatic	
Variables		*p*‐Value	AOR (95% CI)	*p*‐Value	AOR (95% CI)
Division (ref: Barisal)	Chittagong	0.91	0.99 (0.86–1.14)	**0.01**	**1.95 (1.20–3.18)**
	Dhaka	**<0.001**	**0.81 (0.70–0.93)**	0.09	1.52 (0.93–2.47)
	Khulna	0.27	0.92 (0.80–1.06)	**<0.001**	**2.69 (1.66–4.34)**
	Mymensingh	**<0.001**	**0.81 (0.70–0.93)**	0.73	0.90 (0.51–1.60)
	Rajshahi	**<0.001**	**0.72 (0.62–0.83)**	0.03	1.73 (1.05–2.85)
	Rangpur	**0.03**	**1.17 (1.02–1.35)**	0.99	1.00 (0.57–1.76)
	Sylhet	0.05	0.86 (0.74–1.00)	0.47	1.23 (0.70–2.13)
Area of residence (ref: Urban)	Rural	0.23	0.95 (0.87–1.03)	0.94	0.99 (0.77–1.28)
Religion (ref: Islam)	Other	**<0.001**	**0.73 (0.65–0.83)**	0.25	0.79 (0.53–1.18)
Level of education (ref: No education)	Primary	0.14	0.91 (0.81–1.03)	0.26	1.27 (0.84–1.94)
	Secondary	**0.01**	**0.83 (0.73–0.95)**	0.15	1.38 (0.89–2.14)
	Higher	**0.02**	**0.80 (0.66–0.97)**	0.08	1.61 (0.94–2.78)
Owns a mobile phone (ref: No)	Yes	0.26	1.05 (0.97–1.14)	0.65	0.94 (0.70–1.24)
Media Exposure (ref: Not exposed)	Exposed	**<0.001**	**1.21 (1.11–1.30)**	0.80	1.03 (0.80–1.33)
Ever use of internet (ref: No)	Yes	**<0.001**	**0.87 (0.79–0.96)**	0.40	1.13 (0.85–1.50)
Wealth Index (ref: Poorest)	Poorer	0.21	0.93 (0.83–1.04)	0.68	1.09 (0.73–1.61)
	Middle	0.48	0.96 (0.85–1.08)	0.38	1.19 (0.80–1.77)
	Richer	**0.04**	**0.88 (0.77–0.99)**	0.25	0.77 (0.50–1.20)
	Richest	0.11	0.89 (0.76–1.03)	0.37	1.24 (0.77–1.98)
Number of children (ref: No or one child)	Two children	0.64	1.02 (0.93–1.13)	0.59	0.92 (0.67–1.25)
	Three children	0.11	1.11 (0.98–1.25)	0.54	1.14 (0.76–1.71)
	Four or more children	0.06	1.15 (0.99–1.34)	**0.01**	**1.72 (1.13–2.62)**
Working Status (ref: Not working)	Working	0.28	0.96 (0.88–1.04)	0.69	0.95 (0.74–1.22)
Attitudes toward Violence (ref: No)	Yes	**<0.001**	**1.18 (1.07–1.30)**	0.57	0.91 (0.64–1.28)
Decision‐making ability (ref: Does not make decision)	Makes decision	**<0.001**	**1.26 (1.14–1.40)**	**0.01**	**1.59 (1.10–2.28)**
Husband’s Education level (ref: No education)	Primary	0.55	1.03 (0.93–1.15)	0.49	0.89 (0.64–1.24)
	Secondary	0.19	0.93 (0.83–1.04)	0.15	0.76 (0.52–1.11)
	Higher	0.05	0.86 (0.74–1.00)	0.46	0.84 (0.53–1.33)
Husband’s Working status (ref: Not Working)	Working	0.11	0.85 (0.69–1.04)	0.23	1.67 (0.72–3.86)
Distance to health facility (ref: Big problem)	Not a big problem	**<0.001**	**0.74 (0.69–0.80)**	0.69	0.95 (0.76–1.20)
Age^a^	—	**0.04**	**1.01 (1.00–1.02)**	0.14	1.02 (0.99–1.05)
Age at first cohabitation^a^	—	0.25	1.01 (1.00–1.02)	0.09	1.02 (1.00–1.05)
Husband’s age^a^	—	0.78	1.00 (0.99–1.01)	0.61	0.99 (0.97–1.02)

*Note:* Bold values indicate statistically significant associations (*p* < 0.05) in the multinomial regression model.

^a^Metric variables.

Several factors were associated with a higher likelihood of being symptomatic yet undiagnosed with mental health conditions. Women exposed to media had 21% (95% CI: 1.10–1.32) higher likelihood of being symptomatic but undiagnosed rather than being correctly identified as having or not having mental health issues compared to those not exposed. Women with attitudes of accepting violence had 1.18 times (95% CI: 1.05–1.32) higher odds of being symptomatic but undiagnosed compared to their counterparts. Compared to women not involved in household decision‐making, those involved had 26% (95% CI: 1.12–1.42) higher odds of being symptomatic but undiagnosed relative to being correctly identified.

Conversely, several factors were associated with lower odds of symptomatic but undiagnosed mental health conditions. Compared to Muslim women, non‐Muslim women had 27% (95% CI: 0.62–0.86) lower odds of being symptomatic but undiagnosed. Similarly, women with secondary or higher education had 17% (95% CI: 0.73–0.95) and 20% (95% CI: 0.67–0.96) lower odds of being symptomatic but undiagnosed compared to those with no education. Women who used the internet had 13% (95% CI: 0.78–0.97) lower odds of being symptomatic but undiagnosed compared to being correctly identified in their mental health condition. Finally, women who did not consider distance to a health facility a problem were 26% (95% CI: 0.68–0.82) less likely to be symptomatic but undiagnosed rather than being correctly identified in their mental health condition.

### 3.2. Asymptomatic vs. Correctly Identified

Compared to Barisal, women in Chittagong and Khulna had 1.96 times (95% CI: 1.09–3.50) and 2.69 times (95% CI: 1.46–4.94) higher odds, respectively, of being asymptomatic of their mental health issues, rather than being correctly identified as having or not having mental health issues. Similarly, women with four or more children had 72% (95% CI: 1.12–2.63) higher odds of being asymptomatic of their mental health issues as opposed to women with no or one child. When compared to women not involved in household decision‐making, those involved had 59% (95% CI: 1.08–2.33) higher odds of being asymptomatic compared to being correctly identified with their mental health condition.

Additional multinomial logistic regression analyses were conducted separately for anxiety and depression, with the complete results presented in Tables S3 and S4 of Supporting Information S2, respectively.

## 4. Discussion

This study examined the prevalence and key determinants of self‐reported anxiety, depression, and overall mental health, along with the mental health diagnoses among married women in Bangladesh, using data from the 2022 BDHS. The findings reveal that 32% of women were symptomatic but undiagnosed with a mental health condition and 2.1% remained asymptomatic of their condition, underscoring significant gaps in mental health literacy and access to appropriate healthcare services. It is important to note that the categorisation of ‘symptomatic but undiagnosed’ reflects a mismatch between recent symptom burden and prior clinical recognition rather than a measure of diagnostic accuracy in the clinical sense. Given the structure of the BDHS data, this approach provides a pragmatic population‐level indicator of potential under‐recognition and unmet need for mental health care. These disparities were evident across geographic, sociodemographic, and economic groups, with substantial variation in both mental health outcomes and the accuracy of clinical diagnoses. Accessibility, affordability, digital access to correct information, health literacy, and autonomy were associated with correct diagnosis of mental health. Strong regional inequalities in diagnostic accuracy were observed as participants in Dhaka, Mymensingh, and Rajshahi had lower odds of being symptomatic but undiagnosed their mental health condition, while women from Rangpur had higher odds of being asymptomatic. Furthermore, women in Khulna and Chattogram had higher odds of asymptomatic of their mental health condition.

The study identified significant regional disparities in self‐reported anxiety, depression, overall mental health, and the accuracy of mental health diagnoses, aligning with previous research. Bangladesh is administratively divided into eight divisions that differ markedly in socioeconomic development, urbanisation, and healthcare infrastructure, with Dhaka functioning as the political, economic, and medical centre of the country. Rangpur, located in the north‐west, is among the most socioeconomically disadvantaged divisions, characterised by higher poverty rates and limited access to specialised healthcare services. Women in Rangpur reported higher rates of self‐reported anxiety, depression, poor mental health and undiagnosed symptoms. In contrast, women in Dhaka, Mymensingh and Rajshahi showed lower odds of poor mental health and undiagnosed symptoms. Mymensingh and Rajshahi, while less urbanised than Dhaka, host major public medical universities and referral hospitals, potentially facilitating earlier detection and care. Similarly, previous studies based on local surveys have indicated spatial variations in mental health outcomes [41]. These findings underscore the metropolitan and capital‐centric nature of healthcare access in Bangladesh. Urban centres, particularly Dhaka and Chattogram, show lower average poverty rates but pronounced intra‐urban inequality. As Dhaka serves as the nation’s hub, nearby districts may benefit from the better availability of mental health specialists, which could explain the lower prevalence of undiagnosed cases in these areas. The results highlight the need for higher‐resolution spatial data on mental health and call for interventions that are both geographically targeted and culturally appropriate, ensuring that support systems are responsive to the diverse needs of women across the country.

Affordability and accessibility remain critical to accurate mental health diagnosis. In line with previous research, this study found that women from wealthier households had lower rates of self‐reported anxiety, poor mental health and symptomatic but undiagnosed [42–45]. Financial stability likely offers protection by reducing stress from unmet basic needs, improving access to care and enabling engagement in stress‐relieving activities [43, 46]. However, access to essential psychotropic medications remains extremely limited—fewer than 0.11% receive them for free, while daily out‐of‐pocket costs remain a barrier (5.00 taka/US$0.07 for antipsychotics and 3.00 taka/US$0.04 for antidepressants) [6]. Beyond financial access, physical proximity also plays a key role. Married women who did not report distance to health facilities as a problem were less likely to experience anxiety, depression or be symptomatic but undiagnosed. This supports existing evidence that geographic access enhances timely help‐seeking, accurate diagnosis and continuity of mental health support [47, 48].

Access to accurate information is crucial for understanding mental health. This study found media exposure linked to higher rates of symptomatic but undiagnosed mental health issues, likely due to misinformation, anxiety and social comparison, especially among women with low mental health literacy [44, 49–52]. While mobile phone ownership and media use were also linked to increased reports of poor mental health, the risk of being symptomatic but undiagnosed is the more pressing concern. In contrast, internet use was associated with a reduced risk of both poor mental health and symptomatic but undiagnosed conditions, supporting previous evidence on the value of online mental health resources, peer support, and digital coping tools [53, 54]. These findings point to the need for targeted digital literacy programmes and responsible media practices to improve diagnostic accuracy and promote mental health. The educational attainment plays a critical role in shaping mental health diagnostic outcomes.

Women with higher educational levels were less likely to be symptomatic but undiagnosed, likely due to their better symptom recognition, communication skills, and ability to advocate for appropriate care [34, 55, 56]. In contrast, women with limited education may deprioritise mental health, perceiving it as less urgent than physical conditions. These findings underscore the importance of integrating mental health education into school curricula as an intervention strategy to assess long‐term diagnostic accuracy and reduce mental health disparities.

This study underscores the complex and context‐dependent relationship between women’s empowerment and mental health. Acceptance of IPV was strongly associated with poor mental health and symptomatic but undiagnosed, reflecting the psychological toll of internalised gender norms [34, 44, 57]. Contrary to expectations, women involved in household decision‐making also had higher odds of anxiety, depression and being symptomatic but undiagnosed. In patriarchal settings, increased decision‐making responsibilities may heighten stress, particularly when paired with caregiving burdens and limited support [58, 59].

Previous studies have highlighted the link between gender equality and mental health, showing that women with greater household decision‐making autonomy—such as independently making decisions about their own health care, major household purchases, and visits to family members—are less likely to report probable depression and anxiety symptoms [60]. These findings challenge simplistic notions of empowerment and call for culturally sensitive approaches to gender equity and mental health promotion. Targeted data collection—particularly qualitative insights into women’s autonomy—is essential to unpack the causal pathways linking empowerment, mental health outcomes and diagnostic awareness.

### 4.1. Strength and Limitations

While some studies have examined mental health factors in Bangladesh, this is the first to simultaneously assess the prevalence, cultural and sociodemographic influences and diagnostic accuracy. The study offers key insights into mental health challenges, care‐seeking behaviours and barriers faced by women. These findings can guide stakeholders in creating community strategies to encourage social media use for support. Further research is needed on the role of digital platforms in women’s mental health. This study has several limitations that should be considered when interpreting the findings. First, the use of self‐reported data introduces the possibility of recall bias and social desirability bias, which may affect the accuracy of responses. Second, the study focused exclusively on married women, which limits the generalisability of the results to other population groups. Third, the survey lacked qualitative information, such as the specific content of media exposure or women’s beliefs of patriarchal norms, which could have provided deeper insights into the underlying factors influencing mental health and helped validate the quantitative findings. Fourth, important physiological factors such as pregnancy, postpartum status, and menopausal stage are important, but they were not included in this analysis due to the scope and focus of the study. These factors would be better explored in future research. Lastly, the cross‐sectional nature of the study design prevents any conclusions about causality.

## 5. Conclusion

This study is a critical snapshot depicting the scenario of mental health issues and diagnostic disparities among married women in Bangladesh, highlighting significant geographic, sociodemographic and economic inequalities. The findings of the study echoed the need for evidence‐based and culture‐specific strategies to support women’s mental health, particularly in high‐burden and underserved regions. Interventions should prioritise access to information on mental health, improved health literacy, and the transformation of harmful gender norms that contribute to psychological distress and diagnostic inaccuracies or their lack. While internet use was identified as a protective factor, media exposure and mobile phone use were associated with poorer mental health outcomes, suggesting that digital strategies must be carefully designed. Continuous investment in female education, empowerment, and equitable access to healthcare will remain central to improving mental health outcomes. When thoughtfully implemented, media and digital platforms may also serve as scalable tools for increasing awareness, reducing stigma and strengthening mental healthcare–seeking behaviours at the community level.

## Funding

This research did not receive any specific grant from funding agencies in the public, commercial, or not‐for‐profit sectors. Open access publishing facilitated by Swinburne University of Technology, as part of the Wiley ‐ Swinburne University of Technology agreement via the Council of Australasian University Librarians.

## Disclosure

All authors reviewed and approved the final manuscript.

## Ethics Statement

De‐identified secondary datasets are provided upon request by the DHS program authority. The authors obtained permission from DHS to use the above‐mentioned surveys. Further, this study was approved by the Swinburne University of Technology Human Research Ethics Committee (Approval Number: 20226572‐10707).

## Conflicts of Interest

The authors had no conflict of interest.

## Supporting Information

Additional supporting information can be found online in the Supporting Information section.

## Supporting information


**Supporting Information 1** STROBE checklist for cross‐sectional studies – completed checklist for transparent reporting of the study.

**Supporting Information 2** GAD‐7 and PHQ‐9 scoring criteria, and additional results including: Table S1. Bi‐variate unweighted associations of women’s mental health (self‐reported vs diagnosed) with the selected sociodemographic variables; *N* = 18,605. Table S2. Assessment of multicollinearity among explanatory variables using adjusted generalized variance inflation factors (GVIFs). Table S3. Results of the multinomial regression model for Anxiety, after controlling for sociodemographic covariates and adjusting for survey weights (*N* = 18,605). Table S4. Results of the multinomial regression model for Depression, after controlling for sociodemographic covariates and adjusting for survey weights (*N* = 18,605). Figure S1. Prevalence of mental health diagnosis (Anxiety) across divisions in Bangladesh. Figure S2. Prevalence of mental health diagnosis (Depression) across divisions in Bangladesh.

## Data Availability

The data that support the findings of this study are available from the Demographic and Health Surveys (DHS) program. Authors requested and were granted permission for the project. Data are available from the DHS website http://dhsprogram.com/data/available-datasets with the permission of the Demographic and Health Surveys (DHS) program.

## References

[1] GBD 2019 Mental Disorders Collaborators , Global, Regional, and National Burden of 12 Mental Disorders in 204 Countries and Territories, 1990-2019: A Systematic Analysis for the Global Burden of Disease Study 2019, The Lancet Psychiatry. (2022) 9, no. 2, 137–150, 10.1016/S2215-0366(21)00395-3. 35026139 PMC8776563

[2] Arensman E. , Scott V. , De Leo D. , and Pirkis J. , Suicide and Suicide Prevention From a Global Perspective, Crisis-the Journal of Crisis Intervention and Suicide Prevention. (2020) 41, no. S1, S3–s7, 10.1027/0227-5910/a000664. 32208759

[3] United Nations , With Someone Committing Suicide Every 40 Seconds, Massive Scale-Up of Mental Health Investments Needed, Secretary-General Says in International Observance Message, 2020, Retrieved 24/04/2025 from https://press.un.org/en/2020/sgsm20313.doc.htm.

[4] Wu Y. , Wang L. , and Tao M. , et al.Changing Trends in the Global Burden of Mental Disorders From 1990 to 2019 and Predicted Levels in 25 Years, Epidemiology and Psychiatric Sciences. (2023) 32, 10.1017/S2045796023000756. PMC10689059 37933540

[5] Hossain M. D. , Ahmed H. U. , Chowdhury W. A. , Niessen L. W. , and Alam D. S. , Mental Disorders in Bangladesh: A Systematic Review, BMC Psychiatry. (2014) 14, no. 1, 10.1186/s12888-014-0216-9. PMC4149198 25073970

[6] Hasan M. T. , Anwar T. , and Christopher E. , et al.The Current State of Mental Healthcare in Bangladesh: Part 1 - an Updated Country Profile, BJPsych International. (2021) 18, no. 4, 78–82, 10.1192/bji.2021.41. 34747942 PMC8554893

[7] Kirkbride J. B. , Anglin D. M. , and Colman I. , et al.The Social Determinants of Mental Health and Disorder: Evidence, Prevention and Recommendations, World Psychiatry. (2024) 23, no. 1, 58–90, 10.1002/wps.21160. 38214615 PMC10786006

[8] Kivimäki M. , Batty G. D. , and Pentti J. , et al.Association Between Socioeconomic Status and the Development of Mental and Physical Health Conditions in Adulthood: A Multi-Cohort Study, The Lancet Public Health. (2020) 5, no. 3, e140–e149, 10.1016/S2468-2667(19)30248-8. 32007134

[9] Lund C. , Breen A. , and Flisher A. J. , et al.Poverty and Common Mental Disorders in Low and Middle Income Countries: A Systematic Review, Social Science & Medicine. (2010) 71, no. 3, 517–528, 10.1016/j.socscimed.2010.04.027. 20621748 PMC4991761

[10] Rohde N. , Tang K. K. , Osberg L. , and Rao P. , The Effect of Economic Insecurity on Mental Health: Recent Evidence From Australian Panel Data, Social Science & Medicine. (2016) 151, 250–258, 10.1016/j.socscimed.2015.12.014. 26826683

[11] Akhter S. , Dasvarma G. L. , Saikia U. , and Withers M. H. , Reluctance of Women of Lower socioeconomic Status to use Maternal Healthcare Services–Does Only Cost Matter?, PLOS ONE. (2020) 15, no. 9, 10.1371/journal.pone.0239597. PMC7523962 32991622

[12] Akter M. , Socio-economic Barriers Against Women Equal Right in the Society (a Case of Bangladesh), Open Journal of Social Sciences. (2018) 06, no. 7, 156–166, 10.4236/jss.2018.67012.

[13] Mazumder A. H. , Arafat S. M. Y. , Epidemiology and Burden of Mental Disorders in Bangladesh, Mental Health in Bangladesh: From Bench to Community, 2024, Springer Nature Singapore, 15–38, 10.1007/978-981-97-0610-5_2.

[14] Nuri N. N. , Sarker M. , and Ahmed H. U. , et al.Overall Care-Seeking Pattern and Gender Disparity at a Specialized Mental Hospital in Bangladesh, Materia Socio Medica. (2019) 31, no. 1, 35–39, 10.5455/msm.2019.31.35-39. 31213953 PMC6511372

[15] Southard E. M. L. and Randell H. , Food Insecurity and Women’s Mental Health in the Chitwan Valley of Nepal, SSM - Mental Health. (2024) 6, 10.1016/j.ssmmh.2024.100337. PMC11687357 39742114

[16] Al Azdi Z. , Saif S. I. , and Ashraf Kushal S. , et al.Gender Differences in Mental Health Help-Seeking Behaviour in Bangladesh: Findings From a Cross-Sectional Online Survey, BMJ Open. (2025) 15, no. 5, 10.1136/bmjopen-2024-091933. PMC12056629 40335139

[17] Rafi M. A. , Anika U. S. , Hasan M. T. , and Hossain M. G. , Association Between Women’s Empowerment and Mental Health Help-Seeking Behaviour in Bangladesh: Findings From a Nationally Representative Survey, BMJ Open. (2025) 15, no. 9, 10.1136/bmjopen-2025-099770. PMC12434786 40953863

[18] Koly K. N. , Abdullah R. , and Shammi F. A. , et al.Mental Health and Community-Based Rehabilitation: A Qualitative Description of the Experiences and Perspectives of Service Users and Carers in Bangladesh, Community Mental Health Journal. (2022) 58, no. 1, 52–66, 10.1007/s10597-021-00790-0. 33683536

[19] Saif S. I. , Kushal S. A. , Amin Y. M. , and Al Azdi Z. , Gender Differences in Common Mental Disorders and Inequities in Access to Mental Healthcare Services in Bangladesh, Discover Mental Health. (2025) 6, no. 1, 10.1007/s44192-025-00341-z. PMC12799807 41361019

[20] Haque M. R. , Fazle Rabbi A. M. , Araf F. , Rahman M. M. , and Ahmed G. K. R. , Prevalence of Anxiety and Depression Among Married Women in Bangladesh: An Analysis of Nationally Representative Survey, PLOS Mental Health. (2025) 2, no. 7, 10.1371/journal.pmen.0000387. PMC12798323 41662041

[21] Nuri N. N. , Sarker M. , Ahmed H. U. , Hossain M. D. , Beiersmann C. , and Jahn A. , Pathways to Care of Patients With Mental Health Problems in Bangladesh, International Journal of Mental Health Systems. (2018) 12, no. 1, 1–12, 10.1186/s13033-018-0218-y. 30034515 PMC6052552

[22] Amin M. T. , Ara T. , and Pal B. , et al.Prevalence and Correlates of Anxiety and Depression Among ever-Married Reproductive-Aged Women in Bangladesh: National-Level Insights From the 2022 Bangladesh Demographic and Health Survey, BMC Public Health. (2025) 25, no. 1, 10.1186/s12889-025-22228-y. PMC11938691 40133877

[23] Fitch T. J. , Moran J. , Villanueva G. , Sagiraju H. K. R. , Quadir M. M. , and Alamgir H. , Prevalence and Risk Factors of Depression Among Garment Workers in Bangladesh, International Journal of Social Psychiatry. (2017) 63, no. 3, 244–254, 10.1177/0020764017695576. 28466750

[24] Insan N. , Weke A. , Forrest S. , Rankin J. , and Getinet W. , Social Determinants of Antenatal Depression and Anxiety Among Women in South Asia: A Systematic Review & Meta-Analysis, PLOS ONE. (2022) 17, no. 2, 10.1371/journal.pone.0263760. PMC8827460 35139136

[25] Islam M. A. , Barna S. D. , Raihan H. , Khan M. N. A. , Hossain M. T. , and Pakpour A. H. , Depression and Anxiety Among University Students During the COVID-19 Pandemic in Bangladesh: A Web-Based Cross-Sectional Survey, PLOS ONE. (2020) 15, no. 8, 10.1371/journal.pone.0238162. PMC7449469 32845928

[26] Afreen R. , Surya S. L. , and Jara T. , et al.Enhancing Mental Health Literacy and Care Through Community-Driven Solutions in Rural Bangladesh, Frontiers in Global Women’s Health. (2024) 5, 10.3389/fgwh.2024.1478817. PMC11669598 39726686

[27] Koly K. N. , Tasnim Z. , and Ahmed S. , et al.Mental Healthcare-Seeking Behavior of Women in Bangladesh: Content Analysis of a Social Media Platform, BMC Psychiatry. (2022) 22, no. 1, 10.1186/s12888-022-04414-z. PMC9760542 36529761

[28] National Institute of Population Research and Training (NIPORT) and ICF , Bangladesh Demographic and Health Survey 2022: Final Report, 2024, https://www.dhsprogram.com/pubs/pdf/FR386/FR386.pdf.

[29] Spitzer R. L. , Kroenke K. , Williams J. B. , and Löwe B. , A Brief Measure for Assessing Generalized Anxiety Disorder: The GAD-7, Archives of Internal Medicine. (2006) 166, no. 10, 1092–1097, 10.1001/archinte.166.10.1092. 16717171

[30] Kroenke K. , Spitzer R. L. , and Williams J. B. W. , The PHQ-9: Validity of a Brief Depression Severity Measure, Journal of General Internal Medicine. (2001) 16, no. 9, 606–613, 10.1046/j.1525-1497.2001.016009606.x. 11556941 PMC1495268

[31] Cohen D. A. , Scribner R. A. , and Farley T. A. , A Structural Model of Health Behavior: A Pragmatic Approach to Explain and Influence Health Behaviors at the Population Level, Preventive Medicine. (2000) 30, no. 2, 146–154, 10.1006/pmed.1999.0609. 10656842

[32] Krieger N. , Epidemiology and the web of Causation: Has Anyone Seen the Spider?, Social Science & Medicine. (1994) 39, no. 7, 887–903, 10.1016/0277-9536(94)90202-X. 7992123

[33] Bandura A. , Health Promotion by Social Cognitive Means, Health Education & Behavior. (2004) 31, no. 2, 143–164, 10.1177/1090198104263660. 15090118

[34] Gunarathne L. , Nedeljkovic M. , Apputhurai P. , and Bhowmik J. , Impact of Intimate Partner Violence on Mental Health Among Married Women in Sri Lanka: A Study Based on Women’s Wellbeing Survey-2019, Journal of Public Health. (2024) 46, no. 3, e410–e418, 10.1093/pubmed/fdae082. 38852948 PMC11358628

[35] Kabir R. , Majumder M. A. , and Arafat S. M. Y. , et al.Impact of Intimate Partner Violence on Ever Married Women and Utilization of Antenatal Care Services in Tanzania, Journal of College of Medical Sciences-Nepal. (2018) 14, no. 1, 7–13, 10.3126/jcmsn.v14i1.17802.

[36] Sharma K. K. , Vatsa M. , Kalaivani M. , and Bhardwaj D. , Mental Health Effects of Domestic Violence Against Women in Delhi: A Community-Based Study, Journal of Family Medicine and Primary Care. (2019) 8, no. 7, 2522–2527, 10.4103/jfmpc.jfmpc_427_19. PMC6691463 31463288

[37] Vives-Cases C. , Ruiz-Cantero M. T. , Escriba-Aguir V. , and Miralles J. J. , The Effect of Intimate Partner Violence and Other Forms of Violence Against Women on Health, Journal of Public Health. (2011) 33, no. 1, 15–21, 10.1093/pubmed/fdq101. 21196478

[38] Bhowmik J. , Biswas R. K. , Ananna N. , and Wilunda C. , Women’s Education and Coverage of Skilled Birth Attendance: An Assessment of Sustainable Development Goal 3.1 in the South and Southeast Asian Region, PLOS ONE. (2020) 15, no. 4, 10.1371/journal.pone.0231489. PMC7173780 32315328

[39] Gamage S. , Biswas R. K. , and Bhowmik J. , Health Awareness and Skilled Birth Attendance: An Assessment of Sustainable Development Goal 3.1 in South and South-East Asia, Midwifery. (2022) 115, 10.1016/j.midw.2022.103480. 36116336

[40] Fox J. and Monette G. , Generalized Collinearity Diagnostics, Journal of the American Statistical Association. (1992) 87, no. 417, 178–183, 10.1080/01621459.1992.10475190.

[41] Rozario L. G. and Islam S. , Scenario of Mental Health in Bangladesh: A Signature Glimpse, Dhaka University Journal of Biological Sciences. (2022) 405–416, 405–416, 10.3329/dujbs.v30i3.59033.

[42] Ettman C. K. , Cohen G. H. , and Galea S. , Is Wealth Associated With Depressive Symptoms in the United States?, Annals of Epidemiology. (2020) 43, 25–31.e1, 10.1016/j.annepidem.2020.02.001. 32147320 PMC7891298

[43] Ryu S. and Fan L. , The Relationship Between Financial Worries and Psychological Distress Among U.S, Journal of Family and Economic Issues. (2023) 44, no. 1, 16–33, 10.1007/s10834-022-09820-9. 35125855 PMC8806009

[44] Sheoti I. H. , Shezuti S. S. , and Alam M. Z. , Factors Associated With Anxiety and Depression Among Reproductive-Aged Women in Bangladesh: Insights From the 2022 DHS, medRxiv. (2024) 10.1101/2024.12.30.24319793.

[45] Wang X. , Zhao G. , Di J. , Wang L. , and Zhang X. , Prevalence and Risk Factors for Depressive and Anxiety Symptoms in Middle-Aged Chinese Women: A Community-Based Cross-Sectional Study, BMC Women’s Health. (2022) 22, no. 1, 10.1186/s12905-022-01908-6. PMC9338469 35906641

[46] Malak N. and Arshad A. , Impact of Financial Inclusion on Healthcare Access: Evidence From Developing Countries, Journal of Economic and Administrative Sciences. (2024) 42, no. 3, 717–731, 10.1108/JEAS-02-2024-0057.

[47] Chen Y.-Y. , Wong G. H. Y. , and Lum T. Y. , et al.Neighborhood Support Network, Perceived Proximity to Community Facilities and Depressive Symptoms Among Low Socioeconomic Status Chinese Elders, Aging & Mental Health. (2016) 20, no. 4, 423–431, 10.1080/13607863.2015.1018867. 25775108

[48] Tomita A. , Vandormael A. M. , Cuadros D. , Slotow R. , Tanser F. , and Burns J. K. , Proximity to Healthcare Clinic and Depression Risk in South Africa: Geospatial Evidence From a Nationally Representative Longitudinal Study, Social Psychiatry and Psychiatric Epidemiology. (2017) 52, no. 8, 1023–1030, 10.1007/s00127-017-1369-x. 28299376 PMC5534383

[49] Bizzotto N. , de Bruijn G.-J. , and Schulz P. J. , Buffering Against Exposure to Mental Health Misinformation in Online Communities on Facebook: The Interplay of Depression Literacy and Expert Moderation, BMC Public Health. (2023) 23, no. 1, 10.1186/s12889-023-16404-1. PMC10436646 37596592

[50] Dietrich J. J. , Otwombe K. , and Pakhomova T. E. , et al.High Cellphone use Associated With Greater Risk of Depression Among Young Women Aged 15-24 Years in Soweto and Durban, South Africa, Global Health Action. (2021) 14, no. 1, 10.1080/16549716.2021.1936792. PMC8405067 34431754

[51] Primack B. A. , Swanier B. , Georgiopoulos A. M. , Land S. R. , and Fine M. J. , Association Between Media use in Adolescence and Depression in Young Adulthood: A Longitudinal Study, Archives of General Psychiatry. (2009) 66, no. 2, 181–188, 10.1001/archgenpsychiatry.2008.532. 19188540 PMC3004674

[52] Sabar S. , Dzulkalnine N. , and Khir M. M. , The Impact of Social Media on Mental Health of Young Adults: A Literature Review, Information Management and Business Review. (2024) 16, no. 3S(I)a, 447–460, 10.22610/imbr.v16i3S(I)a.4146.

[53] Bucci S. , Schwannauer M. , and Berry N. , The Digital Revolution and Its Impact on Mental Health Care, Psychology and Psychotherapy: Theory, Research and Practice. (2019) 92, no. 2, 277–297, 10.1111/papt.12222. 30924316

[54] Naslund J. A. , Bondre A. , Torous J. , and Aschbrenner K. A. , Social Media and Mental Health: Benefits, Risks, and Opportunities for Research and Practice, Journal of Technology in Behavioral Science. (2020) 5, no. 3, 245–257, 10.1007/s41347-020-00134-x. 33415185 PMC7785056

[55] Chlapecka A. , Wolfová K. , Fryčová B. , and Cermakova P. , Educational Attainment and Anxiety in Middle-Aged and Older Europeans, Scientific Reports. (2023) 13, no. 1, 10.1038/s41598-023-40196-4. PMC10432412 37587157

[56] Irshad Ahmad R. , Dr T. S. , and Shabir Ahmad D. , Women’s Access to Education and Its Impact on Their Empowerment: A Comprehensive Review, MORFAI JOURNAL. (2022) 1, no. 2, 446–450, 10.54443/morfai.v1i2.760.

[57] Ibala R.-M. , Seff I. , and Stark L. , Attitudinal Acceptance of Intimate Partner Violence and Mental Health Outcomes for Female Survivors in Sub-Saharan Africa, International Journal of Environmental Research and Public Health. (2021) 18, no. 10, 10.3390/ijerph18105099. PMC8150836 34065818

[58] Raza S. , Banik R. , and Noor S. T. A. , et al.Anxiety and Depression Among Reproductive-Aged Women in Bangladesh: Burden, Determinants, and Care-Seeking Practices Based on a Nationally Representative Demographic and Health Survey, Archives of Women’s Mental Health. (2025) 28, 1125–1141, 10.1007/s00737-025-01564-3. PMC12436553 39964560

[59] Shawon M. S. R. , Hossain F. B. , Ahmed R. , Poly I. J. , Hasan M. , and Rahman M. R. , Role of Women Empowerment on Mental Health Problems and Care-Seeking Behavior Among Married Women in Nepal: Secondary Analysis of Nationally Representative Data, Archives of Women’s Mental Health. (2024) 27, no. 4, 527–536, 10.1007/s00737-024-01433-5. PMC11230993 38315185

[60] Antabe R. , Antabe G. , Sano Y. , and Pienaah C. K. A. , Women’s Household Decision-Making Autonomy and Mental Health Outcomes in Mozambique, Cambridge Prisms: Global Mental Health. (2025) 12, 10.1017/gmh.2025.29. PMC12037352 40303963

