# Supplementary material for: Mental Health Crisis Among Married Women in Bangladesh: Risk Factors and the Role of Misdiagnosis
Source: Depress Anxiety. 2026 Aug 30;2026:5515696. doi: 10.1155/da/5515696 (PMC13527382; doi:10.1155/da/5515696)
Supplement: Supplementary file 1 [file DA-2026-5515696-s001.docx]

**Supplemental Methods**

*S1. GAD-7 and PHQ-9 Item Descriptions and Classification Criteria*

The GAD-7 scale consists of seven items assessing anxiety symptoms, including feeling nervous, anxious, or on edge; not being able to stop or control worrying; worrying too much about different things; trouble relaxing; being so restless that it is hard to sit still; becoming easily annoyed or irritable; and feeling afraid as if something awful might happen (Spitzer et al., 2006). Each item is rated on a four-point Likert scale (0 = Not at all, 1 = Several days, 2 = More than half the days, 3 = Nearly every day), generating a total score ranging from 0 to 21. The severity of anxiety is categorised as minimal (0–4), mild (5–9), moderate (10–14), and severe (15–21) (Spitzer et al., 2006). In this study, self-reported anxiety was classified as 'no anxiety' for women in the minimal category, and as 'anxiety' for those in the mild, moderate or severe categories.

The PHQ-9 scale assesses depressive symptoms through nine items including little interest or pleasure in doing things; feeling down, depressed, or hopeless; trouble falling asleep or staying asleep or sleeping too much; feeling tired or having little energy; poor appetite or overeating; feeling bad about yourself or that you are a failure or have let yourself or your family down; trouble concentrating on things such as reading the newspaper or watching television; moving or speaking so slowly that other people could have noticed or the opposite; and thoughts that you would be better off dead or of hurting yourself in some way (Kroenke et al., 2001). Similar to the GAD-7, PHQ-9 responses were rated on a four-point Likert scale, with a total score ranging from 0 to 27. Depression severity is categorised into no depression (0–4), mild (5–9), moderate (10–14), moderately severe (15–19), and severe (20–27) (Kroenke et al., 2001). In this study, self-reported depression was classified as 'no depression' for woman in the no depression (minimal) category, and as 'depression' for those in the mild, moderate, moderately severe or severe categories.

**Supplemental Results**

*S2. Post hoc comparisons following ANOVA (related to Table 2 in the main document).*

Homogeneity of variances was assessed using Levene's test before conducting ANOVA. As the assumption of equal variances was violated for respondent's current age (*p* = 0.049), Games–Howell post hoc comparisons were performed. Women in the correctly identified group were significantly younger than those in the self-reported only group (mean difference = 1.45 years, *p* < 0.001) and the diagnosed only group (mean difference = 1.86 years, *p* < 0.001). No significant difference was observed between the self-reported only and diagnosed only groups (*p* = 0.649).

For age at first cohabitation and husband's age, Levene's tests indicated homogeneity of variances (*p* = 0.162 and *p* = 0.448, respectively). Therefore, Tukey's HSD post hoc comparisons were applied. Women in the self-reported only group had a significantly younger age at first cohabitation than those in the correctly identified group (mean difference = 0.15 years, p = 0.021) and the diagnosed only group (mean difference = 0.46 years, p = 0.039). No significant difference was observed between the correctly identified and diagnosed only groups (p = 0.224). Husbands of women in the correctly identified group were significantly younger than those in the self-reported only group (mean difference = 1.55 years, p < 0.001) and the diagnosed only group (mean difference = 1.76 years, p = 0.003). No significant difference was observed between the self-reported only and diagnosed only groups (p = 0.918).

**Table S1.** Bi-variate unweighted associations of women’s mental health (self-reported vs diagnosed) with the selected socio-demographic variables; N=18,605.

| Variables | Self-reported vs Diagnosed Anxiety | | | Self-reported vs Diagnosed Depression | | |
| --- | --- | --- | --- | --- | --- | --- |
|  | Correctly identified  (N=14043, 75.5%) | Symptomatic but Undiagnosed  (N=4035, 21.7%) | Asymptomatic (N=527, 2.8%) | Correctly identified  (N=13526, 72.7%) | Symptomatic but Undiagnosed (N=4784, 25.7%) | Asymptomatic (N=295, 1.6%) |
|  | N (%) | N (%) | N (%) | N (%) | N (%) | N (%) |
| **Division** | | | |  | | |
| Barisal | 1520 (75.8) | 439 (21.9) | 45 (2.3) | 1437 (71.7) | 530 (26.4) | 37 (1.9) |
| Chittagong | 2042 (74.1) | 631 (22.9) | 84 (3.0) | 1947 (70.6) | 779 (28.3) | 31 (1.1) |
| Dhaka | 2197 (78.8) | 525 (18.8) | 66 (2.4) | 2079 (74.6) | 669 (24) | 40 (1.4) |
| Khulna | 1842 (75.4) | 508 (20.8) | 94 (3.8) | 1727 (70.7) | 658 (26.9) | 59 (2.4) |
| Mymensingh | 1542 (76.2) | 435 (21.5) | 46 (2.3) | 1542 (76.2) | 458 (22.6) | 23 (1.1) |
| Rajshahi | 1857 (77.2) | 455 (18.9) | 93 (3.9) | 1759 (73.1) | 576 (24) | 70 (2.9) |
| Rangpur | 1578 (70.9) | 593 (26.6) | 56 (2.5) | 1570 (70.5) | 634 (28.5) | 23 (1) |
| Sylhet | 1465 (74.9) | 449 (22.9) | 43 (2.2) | 1465 (74.9) | 480 (24.5) | 12 (0.6) |
| p-value (Cramer’s V) | <0.001 (0.049) | | | <0.001 (0.052) | | |
| **Religion** | | | |  | | |
| Islam | 12521 (75.1) | 3679 (22.1) | 475 (2.8) | 12036 (72.2) | 4363 (26.2) | 276 (1.7) |
| Other | 1522 (78.9) | 356 (18.4) | 52 (2.7) | 1490 (77.2) | 421 (21.8) | 19 (1.0) |
| p-value (Cramer’s V) | <0.001 (0.027) | | | <0.001 (0.036) | | |
| **Area of residence** | | | |  | | |
| Urban | 4915 (75.8) | 1388 (21.4) | 182 (2.8) | 4726 (72.9) | 1669 (25.7) | 90 (1.4) |
| Rural | 9128 (75.3) | 2647 (21.8) | 345 (2.8) | 8800 (72.6) | 3115 (25.7) | 205 (1.7) |
| p-value (Cramer’s V) | 0.770 (0.005) | | | 0.287 (0.012) | | |
| **Level of education** | | | |  | | |
| No education | 1607 (67.7) | 698 (29.4) | 68 (2.9) | 1627 (68.6) | 714 (30.1) | 32 (1.3) |
| Primary | 3487 (72.7) | 1184 (24.7) | 128 (2.7) | 3384 (70.5) | 1342 (28) | 73 (1.5) |
| Secondary | 6691 (77.5) | 1701 (19.7) | 238 (2.8) | 6378 (73.9) | 2113 (24.5) | 139 (1.6) |
| Higher | 2258 (80.6) | 452 (16.1) | 93 (3.3) | 2137 (76.2) | 615 (21.9) | 51 (1.8) |
| p-value (Cramer’s V) | <0.001 (0.070) | | | <0.001 (0.042) | | |
| **Owns a mobile telephone** | | | |  | | |
| No | 4283 (74.9) | 1288 (22.5) | 147 (2.6) | 4082 (71.4) | 1559 (27.3) | 77 (1.3) |
| Yes | 9760 (75.7) | 2747 (21.3) | 380 (2.9) | 9444 (73.3) | 3225 (25) | 218 (1.7) |
| p-value (Cramer’s V) | 0.081 (0.016) | | | 0.002 (0.026) | | |
| **Media Exposure** | | | |  | | |
| Not exposed | 5731 (74.7) | 1739 (22.7) | 207 (2.7) | 5637 (73.4) | 1925 (25.1) | 115 (1.5) |
| Exposed | 8312 (76.1) | 2296 (21) | 320 (2.9) | 7889 (72.2) | 2859 (26.2) | 180 (1.6) |
| p-value (Cramer’s V) | 0.022 (0.020) | | | 0.161 (0.014) | | |
| **Ever use of internet** | | | |  | | |
| No | 9646 (73.7) | 3086 (23.6) | 363 (2.8) | 9347 (71.4) | 3529 (26.9) | 219 (1.7) |
| Yes | 4397 (79.8) | 949 (17.2) | 164 (3) | 4179 (75.8) | 1255 (22.8) | 76 (1.4) |
| p-value (Cramer’s V) | <0.001 (0.070) | | | <0.001 (0.046) | | |
| **Wealth Index** | | | |  | | |
| Poorest | 2392 (72.5) | 836 (25.3) | 72 (2.2) | 2361 (71.5) | 902 (27.3) | 37 (1.1) |
| Poorer | 2672 (73.4) | 857 (23.6) | 109 (3) | 2591 (71.2) | 978 (26.9) | 69 (1.9) |
| Middle | 2782 (75.3) | 798 (21.6) | 113 (3.1) | 2667 (72.2) | 967 (26.2) | 59 (1.6) |
| Richer | 2959 (76.4) | 809 (20.9) | 104 (2.7) | 2838 (73.3) | 980 (25.3) | 54 (1.4) |
| Richest | 3238 (78.9) | 735 (17.9) | 129 (3.1) | 3069 (74.8) | 957 (23.3) | 76 (1.9) |
| p-value (Cramer’s V) | <0.001 (0.045) | | | <0.001 (0.028) | | |
| **Number of children** | | | |  | | |
| No or one | 4837 (79.8) | 1075 (17.7) | 150 (2.5) | 4587 (75.7) | 1393 (23) | 82 (1.4) |
| Two children | 4649 (76.3) | 1282 (21) | 162 (2.7) | 4463 (73.2) | 1534 (25.2) | 96 (1.6) |
| Three children | 2725 (72.5) | 921 (24.5) | 115 (3.1) | 2653 (70.5) | 1038 (27.6) | 70 (1.9) |
| 4 or more | 1832 (68.1) | 757 (28.2) | 100 (3.7) | 1823 (67.8) | 819 (30.5) | 47 (1.7) |
| p-value (Cramer’s V) | <0.001 (0.066) | | | <0.001 (0.043) | | |
| **Working Status** | | | |  | | |
| Not Working | 9875 (76.1) | 2758 (21.2) | 349 (2.7) | 9514 (73.3) | 3263 (25.1) | 205 (1.6) |
| Working | 4168 (74.1) | 1277 (22.7) | 178 (3.2) | 4012 (71.3) | 1521 (27) | 90 (1.6) |
| p-value (Cramer’s V) | 0.011 (0.022) | | | 0.022 (0.020) | | |
| **Attitudes toward Violence** | | | |  | | |
| No | 12294 (76.2) | 3383 (21) | 452 (2.8) | 11810 (73.2) | 4061 (25.2) | 258 (1.6) |
| Yes | 1749 (70.6) | 652 (26.3) | 75 (3) | 1716 (69.3) | 723 (29.2) | 37 (1.5) |
| p-value (Cramer’s V) | <0.001 (0.045) | | | <0.001 (0.031) | | |
| **Decision-making ability** | | | |  | | |
| Does not make decisions | 2199 (79.3) | 513 (18.5) | 60 (2.2) | 2118 (76.4) | 616 (22.2) | 38 (1.4) |
| Makes decisions | 11844 (74.8) | 3522 (22.2) | 467 (2.9) | 11408 (72.1) | 4168 (26.3) | 257 (1.6) |
| p-value (Cramer’s V) | <0.001 (0.038) | | | <0.001 (0.035) | | |
| **Husband’s Education level** | | | |  | | |
| No education | 2749 (70.2) | 1058 (27) | 111 (2.8) | 2715 (69.3) | 1125 (28.7) | 78 (2) |
| Primary | 3858 (73.9) | 1212 (23.2) | 148 (2.8) | 3772 (72.3) | 1390 (26.6) | 56 (1.1) |
| Secondary | 4639 (77.6) | 1193 (19.9) | 148 (2.5) | 4397 (73.5) | 1495 (25) | 88 (1.5) |
| Higher | 2797 (80.2) | 572 (16.4) | 120 (3.4) | 2642 (75.7) | 774 (22.2) | 73 (2.1) |
| p-value (Cramer’s V) | <0.001 (0.063) | | | <0.001 (0.041) | | |
| **Husband’s Working status** | | | |  | | |
| Not Working | 384 (68.6) | 165 (29.5) | 11 (1.9) | 381 (68.0) | 174 (31.1) | 5 (0.9) |
| Working | 13659 (75.7) | 3870 (21.4) | 516 (2.9) | 13145 (72.8) | 4610 (25.5) | 290 (1.6) |
| p-value (Cramer’s V) | <0.001 (0.034) | | | 0.007 (0.023) | | |
| **Distance to health facility** | | | |  | | |
| Big problem | 6134 (72.6) | 2085 (24.7) | 234 (2.8) | 5887 (69.6) | 2412 (28.5) | 154 (1.8) |
| Not a big problem | 7909 (77.9) | 1950 (19.2) | 293 (2.9) | 7639 (75.2) | 2372 (23.4) | 141 (1.4) |
| p-value (Cramer’s V) | <0.001 (0.066) | | | <0.001 (0.063) | | |
|  | Mean (SD) | Mean (SD) | Mean (SD) | Mean (SD) | Mean (SD) | Mean (SD) |
| **Age*** | 31.24 (8.85) | 33.25 (8.58) | 33.52 (8.55) | 31.4 (8.82) | 32.64 (8.77) | 32.85 (8.69) |
| p-value (η^2^) | <0.001 (0.010) | | | <0.001(0.004) | | |
| **Age at first cohabitation*** | 16.79 (3.59) | 16.59 (3.78) | 16.95 (3.5) | 16.79 (3.58) | 16.66 (3.78) | 16.61 (3.5) |
| p-value (η^2^) | 0.004 (0.001) | | | 0.077 (0.000) | | |
| **Husband’s age*** | 39.17 (10.58) | 41.35 (10.69) | 41.35 (10.15) | 39.34 (10.58) | 40.68 (10.74) | 40.61 (10.12) |
| p-value (η^2^) | <0.001 (0.008) | | | <0.001 (0.003) | | |

*Metric variables

Correctly Identified

Symptomatic but undiagnosed

Asymptomatic

Correctly Identified (%)

Symptomatic but undiagnosed (%)

Asymptomatic (%)

Figure S1. Prevalence of mental health diagnosis (Anxiety) across divisions in Bangladesh.

Correctly Identified

Symptomatic but undiagnosed

Asymptomatic

Correctly Identified (%)

Symptomatic but undiagnosed (%)

Asymptomatic (%)

Figure S2. Prevalence of mental health diagnosis (Depression) across divisions in Bangladesh.

**Table S2:** Assessment of multicollinearity among explanatory variables using adjusted generalized variance inflation factors (GVIFs).

| **Variable** | **Adjusted GVIF (GVIF^(1/(2×Df)))** |
| --- | --- |
| Division | 1.03 |
| Area | 1.11 |
| Religion | 1.03 |
| Education | 1.19 |
| Mobile phone ownership | 1.10 |
| Media exposure | 1.11 |
| Internet use | 1.18 |
| Wealth index | 1.09 |
| Number of children | 1.13 |
| Working status | 1.04 |
| Attitude toward IPV | 1.01 |
| Decision-making ability | 1.02 |
| Husband's education | 1.15 |
| Husband's working status | 1.02 |
| Distance to health facility | 1.02 |
| Respondent age | **2.32** |
| Age at first cohabitation | 1.17 |
| Husband's age | 2.10 |

**Table S3:** Results of the multinomial regression model for Anxiety, after controlling for socio-demographic covariates and adjusting for survey weights (N=18,605).

| Mental Health Diagnosis (ref: Correctly identified) | | Symptomatic but Undiagnosed | | Asymptomatic | |
| --- | --- | --- | --- | --- | --- |
| Variables | | **p-value** | **AOR (95% CI)** | **p-value** | **AOR (95% CI)** |
| **Division**  (ref: Barisal) | Chittagong | 0.545 | 1.08 (0.85 - 1.36) | 0.115 | 1.50 (0.91 - 2.50) |
|  | Dhaka | 0.155 | 0.86 (0.70 - 1.06) | 0.856 | 0.96 (0.58 - 1.56) |
|  | Khulna | 0.982 | 1.00 (0.80 - 1.26) | **0.023** | **1.76 (1.08 - 2.86)** |
|  | Mymensingh | 0.698 | 0.95 (0.76 - 1.21) | 0.936 | 0.97 (0.52 - 1.83) |
|  | Rajshahi | 0.084 | 0.82 (0.65 - 1.03) | 0.078 | 1.58 (0.95 - 2.63) |
|  | Rangpur | **0.001** | **1.45 (1.15 - 1.82)** | 0.518 | 1.23 (0.66 - 2.27) |
|  | Sylhet | 0.849 | 1.02 (0.80 - 1.31) | 0.764 | 1.09 (0.63 - 1.87) |
| **Area of residence**  (ref: Urban) | Rural | 0.165 | 0.91 (0.79 - 1.04) | 0.880 | 0.98 (0.73 - 1.31) |
| **Religion**  (ref: Islam) | Other | **<0.001** | **0.72 (0.60 - 0.86)** | 0.688 | 0.91 (0.58 - 1.44) |
| **Level of education**  (ref: No education) | Primary | 0.079 | 0.89 (0.78 - 1.01) | 0.763 | 1.06 (0.73 - 1.52) |
|  | Secondary | **0.018** | **0.83 (0.72 - 0.97)** | 0.535 | 1.13 (0.77 - 1.67) |
|  | Higher | **0.021** | **0.78 (0.63 - 0.96)** | 0.406 | 1.25 (0.73 - 2.14) |
| **Owns a mobile phone**  (ref: No) | Yes | **0.007** | **1.14 (1.04 - 1.24)** | 0.541 | 1.08 (0.84 - 1.39) |
| **Media Exposure**  (ref: Not exposed) | Exposed | **0.019** | **1.13 (1.02 - 1.24)** | 0.934 | 1.01 (0.81 - 1.26) |
| **Ever use of internet**  (ref: No) | Yes | **0.029** | **0.87 (0.76 - 0.99)** | 0.453 | 1.11 (0.84 - 1.46) |
| **Wealth Index**  (ref: Poorest) | Poorer | 0.384 | 0.94 (0.83 - 1.08) | 0.089 | 1.36 (0.95 - 1.95) |
|  | Middle | 0.088 | 0.89 (0.78 - 1.02) | 0.153 | 1.31 (0.90 - 1.91) |
|  | Richer | **0.047** | **0.86 (0.74 – 1.00)** | 0.994 | 1.00 (0.67 - 1.50) |
|  | Richest | **0.014** | **0.80 (0.68 - 0.96)** | 0.266 | 1.28 (0.83 - 1.97) |
| **Number of children**  (ref: No or one child) | Two children | 0.541 | 1.04 (0.92 - 1.17) | 0.835 | 1.03 (0.78 - 1.36) |
|  | Three children | 0.229 | 1.09 (0.95 - 1.25) | 0.528 | 1.12 (0.78 - 1.62) |
|  | 4 or more children | 0.124 | 1.14 (0.97 - 1.34) | **0.020** | **1.59 (1.08 - 2.36)** |
| **Working Status**  (ref: Not working) | Working | 0.338 | 0.95 (0.86 - 1.05) | 0.880 | 0.98 (0.79 - 1.23) |
| **Attitudes toward Violence**  (ref: No) | Yes | **<0.001** | **1.27 (1.12 - 1.45)** | 0.251 | 1.20 (0.88 - 1.64) |
| **Decision-making ability**  (ref: Does not make decisions) | Make decisions | **0.006** | **1.21 (1.06 - 1.38)** | **0.038** | **1.40 (1.02 - 1.93)** |
| **Husband’s Education level**  (ref: No education) | Primary | 0.837 | 1.01 (0.91 - 1.13) | 0.791 | 0.96 (0.73 - 1.28) |
|  | Secondary | 0.076 | 0.89 (0.78 - 1.01) | 0.423 | 0.86 (0.60 - 1.24) |
|  | Higher | **0.013** | **0.79 (0.66 - 0.95)** | 0.962 | 0.99 (0.66 - 1.48) |
| **Husband’s Working status**  (ref: Not Working) | Working | **0.003** | **0.70 (0.56 - 0.89)** | 0.427 | 1.30 (0.68 - 2.51) |
| **Distance to health facility**  (ref: Big problem) | Not a big problem | **<0.001** | **0.72 (0.65 - 0.80)** | 0.258 | 0.88 (0.71 - 1.10) |
| **Age*** |  | **<0.001** | **1.02 (1.01 - 1.03)** | **0.029** | **1.03 (1.00 - 1.06)** |
| **Age at first cohabitation*** |  | 0.358 | 1.01 (0.99 - 1.02) | 0.519 | 1.01 (0.98 - 1.03) |
| **Husband’s age*** |  | 0.982 | 1.00 (0.99 - 1.01) | 0.368 | 0.99 (0.97 - 1.01) |

*Metric variables

**Table S4:** Results of the multinomial regression model for Depression, after controlling for socio-demographic covariates and adjusting for survey weights (N=18,605).

| Mental Health Diagnosis (ref: Correctly identified) | | Symptomatic but Undiagnosed | | Asymptomatic | |
| --- | --- | --- | --- | --- | --- |
| Variables | | **p-value** | **AOR (95% CI)** | **p-value** | **AOR (95% CI)** |
| **Division**  (ref: Barisal) | Chittagong | 0.783 | 1.03 (0.83 - 1.29) | 0.062 | 0.56 (0.31 - 1.03) |
|  | Dhaka | 0.060 | 0.84 (0.70 - 1.01) | 0.407 | 0.78 (0.43 - 1.41) |
|  | Khulna | 0.695 | 1.04 (0.87 - 1.24) | 0.341 | 1.31 (0.75 - 2.29) |
|  | Mymensingh | **0.009** | **0.75 (0.60 - 0.93)** | **0.025** | **0.50 (0.28 - 0.92)** |
|  | Rajshahi | **0.023** | **0.81 (0.67 - 0.97)** | 0.117 | 1.54 (0.90 - 2.65) |
|  | Rangpur | 0.140 | 1.16 (0.95 - 1.42) | 0.075 | 0.56 (0.30 - 1.06) |
|  | Sylhet | 0.226 | 0.86 (0.68 - 1.10) | **0.002** | **0.30 (0.14 - 0.65)** |
| **Area of residence**  (ref: Urban) | Rural | 0.554 | 0.96 (0.83 - 1.11) | 0.248 | 1.23 (0.87 - 1.73) |
| **Religion**  (ref: Islam) | Other | **<0.001** | **0.71 (0.59 - 0.85)** | 0.086 | 0.60 (0.33 - 1.08) |
| **Level of education**  (ref: No education) | Primary | 0.595 | 0.96 (0.83 - 1.11) | 0.230 | 1.37 (0.82 - 2.27) |
|  | Secondary | 0.222 | 0.91 (0.78 - 1.06) | 0.158 | 1.48 (0.86 - 2.56) |
|  | Higher | 0.197 | 0.88 (0.72 - 1.07) | 0.303 | 1.45 (0.72 - 2.94) |
| **Owns a mobile phone**  (ref: No) | Yes | 0.468 | 0.97 (0.88 - 1.06) | 0.238 | 1.21 (0.88 - 1.67) |
| **Media Exposure**  (ref: Not exposed) | Exposed | **<0.001** | **1.23 (1.11 - 1.35)** | 0.929 | 0.99 (0.71 - 1.37) |
| **Ever use of internet**  (ref: No) | Yes | 0.150 | 0.92 (0.83 - 1.03) | **0.036** | **0.67 (0.46 - 0.97)** |
| **Wealth Index**  (ref: Poorest) | Poorer | 0.764 | 0.98 (0.85 - 1.12) | **0.014** | **1.71 (1.11 - 2.63)** |
|  | Middle | 0.991 | 1.00 (0.86 - 1.16) | 0.186 | 1.37 (0.86 - 2.19) |
|  | Richer | 0.174 | 0.89 (0.76 - 1.05) | 0.528 | 1.18 (0.70 - 1.99) |
|  | Richest | 0.169 | 0.88 (0.73 - 1.06) | **0.013** | **2.06 (1.16 - 3.65)** |
| **Number of children**  (ref: No or one child) | Two children | 0.814 | 1.01 (0.91 - 1.13) | 0.550 | 1.12 (0.78 - 1.61) |
|  | Three children | 0.230 | 1.09 (0.95 - 1.24) | 0.124 | 1.41 (0.91 - 2.18) |
|  | 4 or more children | 0.046 | 1.17 (1.00 - 1.35) | **0.016** | **1.85 (1.12 - 3.05)** |
| **Working Status**  (ref: Not working) | Working | 0.955 | 1.00 (0.92 - 1.10) | 0.466 | 0.90 (0.68 - 1.20) |
| **Attitudes toward Violence**  (ref: No) | Yes | **0.010** | **1.16 (1.04 - 1.3)** | 0.714 | 1.08 (0.71 - 1.65) |
| **Decision-making ability**  (ref: Does not make decisions) | Makes decisions | **0.001** | **1.24 (1.09 - 1.42)** | 0.181 | 1.34 (0.87 - 2.06) |
| **Husband’s Education level**  (ref: No education) | Primary | 0.732 | 1.02 (0.91 - 1.14) | **0.004** | **0.54 (0.36 - 0.82)** |
|  | Secondary | 0.786 | 0.98 (0.86 - 1.12) | 0.125 | 0.71 (0.46 - 1.10) |
|  | Higher | 0.176 | 0.89 (0.75 - 1.06) | 0.528 | 0.83 (0.47 - 1.47) |
| **Husband’s Working status**  (ref: Not Working) | Working | **0.024** | **0.77 (0.61 - 0.97)** | 0.281 | 1.76 (0.63 - 4.88) |
| **Distance to health facility**  (ref: Big problem) | Not a big problem | **<0.001** | **0.73 (0.67 - 0.81)** | **0.006** | **0.65 (0.47 - 0.88)** |
| **Age*** |  | 0.113 | 1.01 (1.00 - 1.02) | 0.353 | 1.02 (0.98 - 1.05) |
| **Age at first cohabitation*** |  | 0.255 | 1.01 (0.99 - 1.02) | 0.541 | 1.01 (0.98 - 1.05) |
| **Husband’s age*** |  | 0.673 | 1.00 (0.99 - 1.01) | 0.282 | 0.99 (0.96 - 1.01) |

**References**

Kroenke, K., Spitzer, R. L., & Williams, J. B. (2001). The PHQ-9: validity of a brief depression severity measure. *J Gen Intern Med*, *16*(9), 606-613. <https://doi.org/10.1046/j.1525-1497.2001.016009606.x>

Spitzer, R. L., Kroenke, K., Williams, J. B., & Löwe, B. (2006). A brief measure for assessing generalized anxiety disorder: the GAD-7. *Arch Intern Med*, *166*(10), 1092-1097. <https://doi.org/10.1001/archinte.166.10.1092>
